# Perspective Taking and Memory for Self- and Town-Related Information in Male Adolescents and Young Adults

**DOI:** 10.1016/j.cogdev.2023.101356

**Published:** 2023-07

**Authors:** Maximilian Scheuplein, Saz P. Ahmed, Lucy Foulkes, Cait Griffin, Gabriele Chierchia, Sarah-Jayne Blakemore

**Affiliations:** 1 Institute of Cognitive Neuroscience, University College London, Alexandra House, 17-19 Queen Square, London WC1N 3AZ, United Kingdom; 2 Institute of Education and Child Studies, Leiden University, Pieter de la Court building, Wassenaarseweg 52, 2333 AK Leiden, The Netherlands; 3 Department of Psychology, University of Cambridge, Downing Pl, Cambridge CB2 3EB, United Kingdom; 4 Department of Brain and Behavioral Science, University of Pavia, Piazza Botta 6, 27100, Pavia, Italy

**Keywords:** adolescence, social cognitive development, self-concept, self-referential processing, perspective taking, theory of mind

## Abstract

Adolescence is a sensitive period for categorical self-concept development, which affects the ability to take others’ perspectives, which might differ from one’s own, and how self-related information is memorized. Little is known about whether these two processes are related in adolescence. The current study recruited 97 male participants aged 11-35 years. Using a self-referential memory task, we found that younger participants were less prone to recognize previously seen town-related adjectives, compared to self-related adjectives. However, this age-related reduction in recognition bias was unrelated to accurate memory performance. Using the Director task to assess perspective taking, we found an age-related decrease in egocentric biases in perspective taking from adolescence to early adulthood (i.e., perspective taking abilities improved with age). However, there was no evidence that these two processes were related. Overall, our findings suggest that male adolescents display parallel but independent age-related changes in self-referential biases in memory and perspective taking.

## Introduction

Adolescence starts with the onset of puberty and ends when the individual achieves a stable and independent role in society, roughly corresponding to 10-24 years (Dahl, 2004; Sawyer et al., 2018). This transformative period of life is characterized by significant changes in social cognitive behavior and in self-concept domains (Crone & Fuligni, 2020; Erikson, 1968; Sebastian et al., 2008). Defined as how an individual perceives and describes oneself (e.g., “I am athletic”), the categorical self-concept is known to become less concrete and more abstract with increasing age (Cole et al., 2001; Harter, 1990). For example, Montemayor and Eisen (1975) asked individuals aged 9 to 18 years to provide self-descriptions (by answering the question “Who am I?”) and found that children aged 9-11 years were more likely to describe themselves with concrete object labels, such as their home address or possessions, whereas adolescents aged 12-18 years were more likely to describe themselves with more abstract and differentiated trait labels, such as “curious” or “ambitious”.

To explore this shift towards a more abstract categorical self-concept, research has focused on the effect of self-referential memory. It has been proposed that self-related information is processed more deeply and efficiently – therefore leading to higher levels of recall – than other types of information (Craik et al., 1999; Kelley et al., 2002; Klein & Kihlstrom, 1986; Rogers et al., 1977). A typical self-referential memory paradigm presents participants with trait adjectives and asks them to judge the descriptiveness of the adjectives in reference to themselves (e.g., “Does this word describe me?”), in reference to a non-related but familiar other (e.g., “Does this word describe Harry Potter?”) and a control condition (e.g., “How many syllables does this word have?”). A subsequent surprise memory task is then used to assess whether adjectives that have been evaluated in relation to the self are better remembered (i.e., increased memory sensitivity and faster memory recall). Indeed, several studies in children (aged 5-10 years; Cunningham et al., 2014; Halpin et al., 1984; Sui & Zhu, 2005), adolescents (aged 11-18 years; Dégeilh et al., 2015; Henderson et al., 2009; Moses-Payne et al., 2022), and adults (aged 21-82 years; D’Argembeau et al., 2007; Gutchess et al., 2007; Mitchell et al., 2006) suggest that this is generally the case. Behavioral studies have shown that this self-referential memory effect emerges at around four years (Cunningham et al., 2014) and continues to increase from childhood to adolescence, in line with categorical self-concept development (Harter, 1990; Ray et al., 2009). However, the developmental trajectory of this effect beyond adolescence is less well understood.

Age-related changes in self-referential processing may be related to age-related changes in perspective taking. Perspective taking is the ability to take someone else’s perspective and it often involves a trade-off between one’s own perspective (the self-evaluative perspective) and the other’s perspective. Furthermore, perspective taking is a component of the ability to infer other people’s mental states, which is known as “mentalizing” or “theory of mind” (Frith & Frith, 2003; Gallagher & Frith, 2003). While common theory of mind tasks are usually passed by the age of five (Frith & Frith, 2003), the ability to take another person’s perspective in order to guide behavior continues to develop well beyond childhood (Dumontheil et al., 2010; Dumontheil et al., 2012; Symeonidou et al., 2016). This has been shown using the Director task (Dumontheil et al., 2010). In this task, a “director” instructs participants to move objects around a set of shelves. There are two conditions: participants either take account of the director’s perspective (Director condition) or they follow a simple rule without having to take into consideration the director’s perspective (No Director condition). In both conditions, an initial improvement in accuracy was observed between childhood (aged 7-11 years) and mid-adolescence (aged 11-13 years; Dumontheil et al., 2010). Beyond mid-adolescence (aged 14-17 years), no further improvement in accuracy was observed in the No-Director condition, whereas in the Director condition, accuracy continued to improve between adolescence and early adulthood (aged 19-27 years; Dumontheil et al., 2010). These results provide evidence for continued age-related improvements in perspective taking abilities in late adolescence (replicated in Symeonidou et al., 2016).

Self- and other-oriented thinking are suggested to be intertwined developmental processes (Crone & Fuligni, 2020). Simulation and self-projection theories of social cognition have posited that self-knowledge can be used to infer others’ mental states and perspectives. First, Tamir and Mitchell (2010) argued that, in order to take someone else’s perspective, perceivers first use their own introspection as a self-generated “anchor” value to then serially adjust (in a process called “anchoring-and-adjustment”). This account is complimented by research showing that individuals frequently attribute (and sometimes over-attribute) their own thoughts, preferences and perspectives to others (Dinulescu et al., 2021; Sweatman et al., 2022; Todd et al., 2011). In addition, self-referential processing has been proposed to play a role in memory (Dinulescu et al., 2021) and perspective taking, given that both processes involve projecting the self to a different time (in the case of memory) or perspective (Spreng et al., 2009).

Second, research on the looking-glass self (Cooley, 1983) suggests that an individual’s understanding of what they “are like” as a person (i.e., their categorical self-concept) becomes progressively informed by their beliefs about how they are seen by others. Third, autistic adults^1^ (autism is a developmental condition that in some people involves difficulties in mentalizing; (Baron-Cohen et al., 1985)) showed an absent (Toichi et al., 2002) or diminished self-referential memory effect (Lombardo et al., 2007).

Together, previous research suggests that self- and other-oriented processes may be connected. Therefore, the goal of the current study was to assess age-related changes in, and the relationship between, self-referential memory (using the self-referential memory task) and perspective taking (using the Director task) in typically developing male adolescents and young adults.

In addition, this study aimed to corroborate the age trend of the self-referential memory effect within the framework of signal detection theory (SDT) (Locke & Robinson, 2021). One of the advantages of SDT is that it enabled us to differentiate between sensitivity and bias. Sensitivity refers to a cognitive system’s ability to correctly differentiate between signal and noise, such as a tone from background noise, or a new memory trace from older memory traces. Bias refers to decision-related processes that can also affect performance but can be independent of sensitivity. For example, in a memory task in which participants are required to discriminate between previously seen and novel items, two equally insensitive/poor-performing participants might err in opposite directions: one might frequently fail to recognize a previously seen item, displaying many false negatives (i.e., a conservative bias), while the other might frequently report remembering an item that they had actually not seen, displaying many false positives (i.e., a liberal bias). Previous work on the developmental trajectories of self-referential memory effects have adopted the SDT framework (Henderson et al., 2009), but have focused on sensitivity, rather than potential biases. Given that sensitivity and biases refer to different cognitive mechanisms that can vary independently (Rosenstreich & Ruderman, 2016; Wylie et al., 2021), we aimed to fill this gap, with no directional hypotheses.

We examined three hypotheses. First, we investigated self-referential memory across adolescence and adulthood in males. Across age, we expected to find enhanced and faster memory performance (i.e., sensitivity) for self-related (vs. town-related) adjectives (hypothesis 1). Given that the developmental trajectory of the self-referential memory effect is less understood beyond adolescence, we had no prior age-related predictions. In addition, little is known about the development of response biases. Hence, this analysis was treated as exploratory with no directional predictions. Second, we aimed to replicate previous findings demonstrating age-related changes in perspective taking abilities. Participants completed the Director task (adapted from Dumontheil et al., 2010) and we expected adolescents to commit more egocentric errors (ignoring the director’s perspective) than adults (hypothesis 2). Finally, we explored the relationship between self-referential memory and perspective taking abilities. However, given scarce empirical evidence, this analysis was also treated as exploratory with no directional predictions (hypothesis 3). Additionally, we used response times analyses to address whether changes in self-referential processing and perspective taking might be attributed to changes in efficiency or might involve potential speed-accuracy tradeoffs. However, we had no a priori hypotheses.

## Method

### Participants

One hundred and ten males (53 adolescents aged 11.2-17.5 years and 57 adults aged 22.2-35.6 years) took part in this experiment. Previous studies in adolescents and adults using variants of the tasks have found robust group differences with sample sizes of 20-30 participants per age group (Sui & Zhu, 2005; Symeonidou et al., 2016). As we did not know the size of our hypothesized effects, we aimed for a sample size of at least 45 adolescent participants and 45 adult participants, for a total of 90 participants. However, to avoid any arbitrary age-related grouping criteria, all analyses employed age as a continuous variable. Longitudinal research with adolescents, investigating gender differences in perspective taking development, has demonstrated higher levels of perspective taking abilities in 13-18-year-old girls compared with age-matched boys (Van der Graaff et al., 2014). Consequently, we recruited only male participants to test for within-group variation (Keulers et al., 2010; Maccoby, 1998). Data from 12 participants were excluded from all analyses due to parent-reported diagnoses of developmental disorders (two adolescents) and a technical error (one adolescent and nine adults). One additional adult participant was excluded for self-referential memory task performance below chance, leaving a total sample size of *N* = 97 (50 adolescents aged 11.2-17.5 years and 47 adults aged 22.2-35.6 years) for all subsequent analyses. A greater proportion of adolescents self-reported as White (82%) compared to adults (19%), and a lower proportion of adolescents self-reported as Black (0%) and Asian (6%) compared to adults (Black 11%; Asian 64%) (see Table S1 in the supplementary information for detailed participant information).

Adolescents were recruited through social media and from three schools within the Greater London area. Adults were recruited using the university Psychology Department participant database. Testing was either conducted in a laboratory setting at the university or in schools. All included participants spoke English fluently and had no history of psychiatric, developmental, or neurological disorders. Adult participants, and the primary caregiver of the adolescent participants, gave informed consent. The study was approved by the university ethics committee (Project ID Number: 3453/001). Following the completion of the study, participants were debriefed, given the opportunity to ask any questions, and compensated £10 for their time.

### Design and Procedure

The experiment included three main stages. First, participants completed the first part of the self-referential memory task (learning phase), which was followed by the Director task (adapted from Dumontheil et al., 2010). Next, participants completed the second part of the self-referential memory task (recall phase).

#### Self-Referential Memory Task

**Figure 1:**
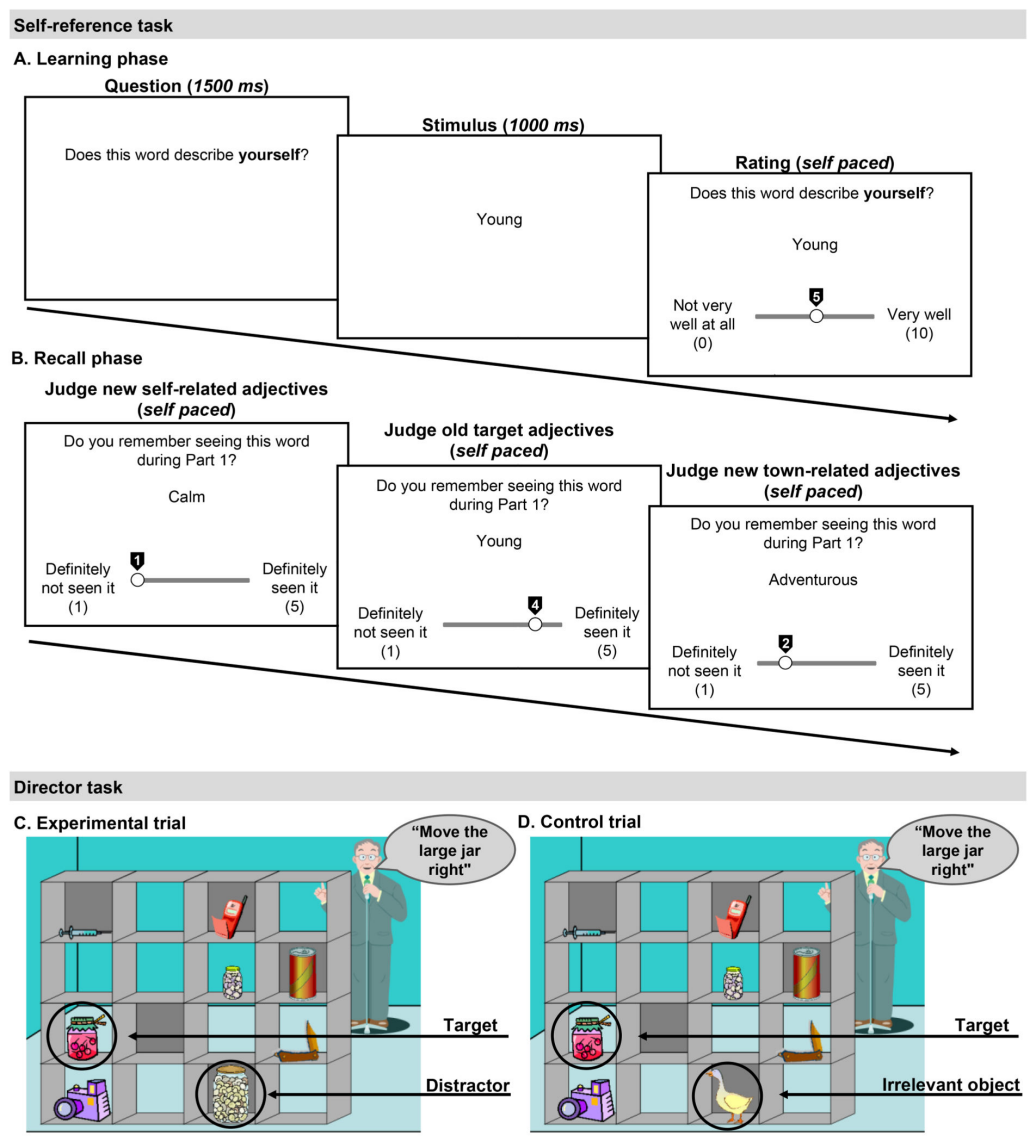
Tasks *Note*. The self-referential memory task (**A-B**). (**A**) Learning phase: participants had to decide whether randomly presented adjectives (20 self-related and 20 town-related) were good descriptions of either themselves (Self condition: “Does this word describe yourself?”) or London (Town condition: “Does this word describe London?”). Responses were given on an 11-point rating scale. (**B**) Recall phase: participants had to judge whether they have already seen one of the randomly presented adjectives (40 target adjectives, 80 distractor adjectives) during the learning phase or not. Responses were given on a 5-point rating scale. The Director task (**C-D**). In this example, participants were verbally instructed by the director to “move the large jar right”. (**C**) During experimental trials, an error would be committed when ignoring the director’s perspective and incorrectly moving the distractor object, which is not visible to the director. In contrast, a correct response would be to move the target object, which is visible to both director and participant. (**D**) During control trials, distractor objects were replaced with irrelevant objects (e.g., the duck).

The self-referential memory task was split into a learning phase and a recall phase (see Figure 1 A-B). At the beginning of each phase participants had the opportunity to ask questions after reading a short introduction. In both phases there were two conditions: self and town. During the learning phase (see Figure 1a), participants had to judge how descriptive a set of self-related adjectives (e.g., “calm”) were of themselves (Self condition: “does this word describe yourself?”) and how descriptive a different set of town-related adjectives (e.g., “adventurous”) were of London (Town condition: “Does this word describe London?”). These questions were presented for 1500 ms before the target adjective was presented for 1000 ms. Participants then indicated how well the adjectives described themselves or London on an 11-point rating scale (0: “not very well at all”, 10: “very well”). This rating was self-paced and used to allow for more nuanced descriptiveness ratings. The task was programmed in Gorilla (https://gorilla.sc/; Anwyl-Irvine et al., 2020) and was presented on 13-inch laptops.

Forty adjectives (20 town-related and 20 self-related) were presented in a randomized order, and it took participants approximately 5 mins to complete this part of the task. All adjectives used in this task were drawn from previous likableness ratings of trait adjectives (Anderson, 1968) and were of positive valence. In addition, self- and town-related words were matched for word length and level of difficulty. The words were piloted with five 10- to 13-year-old males, who were presented with all adjectives and asked to define their meaning. This was to ensure that the youngest participants would understand each word. Based on their responses, 16 adjectives were replaced because they were considered inappropriate or too difficult to understand. A list of all adjectives used in the self-referential memory task can be found in the supplementary information (see Table S2).

During the recall phase (see Figure 1B), which was completed after the Director task (see next section), participants were presented with 120 adjectives comprising all 40 target adjectives from the learning phase and an additional 80 distractor adjectives (40 self-related, 40 town-related), which they had not seen before. Participants were asked whether they remembered seeing the word during the learning phase by indicating how confident they were in their answer on a five-point rating scale (1: “definitely not seen it”, 5: “definitely seen it”). This rating was self-paced and used to isolate variability in confidence judgments. All 120 adjectives were presented in a randomized order. This recall phase took 12-15 mins to complete.

Although both phases had no time restrictions to provide a rating, participants were encouraged to respond as fast as possible, while also thinking carefully about each adjective. Before participants could start one of the phases, they first had to complete two practice trials (one for each condition) to demonstrate that they had understood the instructions.

#### Director Task

Task design and stimuli were taken from the computerized task used in Dumontheil et al. (2010; see Figure 1C-D). The stimuli (48 in total) consisted of sets of 4 x 4 shelves with objects located in half of the 16 slots. Five of the slots had an opaque grey background, which occluded the view of the “director” who stood on the other side of the shelves (i.e., he viewed the shelves from behind). The director gave verbal instructions to move one of the eight objects to a different slot in the shelves. Before completing the task, participants were presented with standardized instructions and example stimuli on a PowerPoint presentation. The instructions required participants to use the director’s visual perspective to determine which objects he could and could not see, and thereby select and move the most appropriate object. To ensure that all instructions were understood correctly, each participant had to point out one object that only he but not the director could see (i.e., any object in occluded slots) and one object that was visible to both director and participant (i.e., objects in clear slots). All participants gave correct responses, indicating that they understood the instructions and were able to describe which objects the director could and could not see. Participants then completed one practice block with three trials. Again, all participants performed this correctly, demonstrating that they understood what was required of them. Similar to the procedure by Dumontheil et al. (2010), they were not given further feedback regarding the requirement to take into account the director’s perspective. The instructions were presented via headphones and participants used the computer mouse to move the object they thought the director was referring to into the appropriate slot on the shelves.

During experimental trials (eight in total), a relevant object could be seen by the participant but not the director (see Figure 1C). Participants were instructed to, for example, “move the large jar right”. They were informed that the director would be referring to the participant’s right or left. A correct response would consider the director’s perspective, and thus the red jar (target object) would be selected and moved to the appropriate slot (i.e., the right slot). An incorrect response would ignore the director’s perspective and thereby move the bottom-most jar (distractor object), which is not visible to the director. In the control trials (eight in total), the object-shelf configurations were identical to that in the experimental trials, except that all relevant objects could be seen by both the participant and the director (see Figure 1D). It was therefore not necessary to consider the director’s perspective to select the correct answer in these trials. During filler trials (32 in total), instructions only referred to objects in clear slots. For example, in Figure 1C the director could ask to “move the knife left”. The order of the experimental, control and filler trials was counterbalanced between participants.

Eight different object-shelf configurations were used, each presented once with an occluded distractor object (experimental trial) and once with an irrelevant object (control trial). In total, this resulted in 16 test blocks of different object-shelf configurations, which were all counterbalanced across participants. Stimuli were presented for 2 s before the first auditory instruction was given. Three auditory instructions were given per stimulus (i.e., one instruction per trial) and each lasted 2.2 s. For example, in Figure 1C the experimental trial would start with the director instructing participants to “move the small jar down”. Within the same shelf-object configuration the director would ask the participant to then “move the scissors up” before finally asking them to “move the large jar right”. After each auditory instruction the participant had 3.6 s to make their response. The task was programmed using E-prime version 2.0 (Psychology Software Tools, Inc.), presented on 13-inch laptops and took approximately 6 mins to complete.

### Statistical Analysis

#### Self-Referential Memory Task

The primary aim of this study was to determine whether self-referential memory and perspective taking were associated with age, and whether they were related. To assess age-related changes in the self-referential memory task, recall phase ratings on the five-point confidence scale (in response to the question “Do you remember seeing this word during Part 1?”) were binarized. Responses above 3 (coded as 1) indicated high confidence that the adjective had been seen during the learning phase, while responses made below 3 (coded as 0) indicated high confidence that the adjective had not been seen. Ratings equal to 3 indicated that participants did not know whether they had seen the presented adjective or not. These ratings were excluded from the analyses (this was the least common response option across participants (6%)). In addition, based on previous literature suggesting that accuracy and meta-cognition (e.g., confidence) are correlated but dissociable constructs (Forsberg et al., 2021; Renner & Renner, 2001), we analyzed how the learning- and recall phase ratings and reaction times changed as a function of age and condition (see supplementary information). Due to poor internet connection, self-referential memory data from one adolescent and two adult participants could not be collected, leaving a total sample size of *N* = 94 for the memory analyses. These analyses employed a signal detection theory framework (Anderson, 2015), which typically includes two measures: d-prime (*d’*) and response biases. *d’* was computed by calculating the difference between the z-transformed probabilities of hits (i.e., the number of target adjectives correctly remembered) and false alarms (FAs; i.e., the number of distractor adjectives falsely thought to be remembered) (*d’* = z(hits) – z(FAs)), separately for each condition (self and town) and each participant. This allowed us to discriminate between the decision signal (hits) and noise (FAs; Stanislaw & Todorov, 1999). The larger the difference between the hit rate and FA rate, the better the participant’s memory sensitivity. That is, the better their ability to correctly discriminate between (target) adjectives that have been previously presented and (distractor) adjectives that had not. In other words, memory sensitivity increases if the hit rate increases and/or FA rate decreases. Additionally, even in the absence of age differences in memory sensitivity, it could still be the case that younger participants are biased toward using one response more often than an alternative response. To test this possibility, we computed a commonly used measure of response bias, *c*, which is typically unaffected by changes in *d’* (Anderson, 2015). The response bias was estimated from the probabilities of hit and FA rates, *c* = -[z(hits) + z(FAs)]/2. An increase in hit *and* FA rates would reflect a liberal criterion (*c* < 0), meaning participants are more likely to report an adjective as present during the learning phase, independently of whether this adjective had actually been seen or not. In contrast, a more conservative criterion (*c* > 0) would indicate that participants were less likely to report an adjective as present during the learning phase, resulting in less FAs, but more misses. The absolute value of *c* provides an indication of the strength of a participant’s bias. That is, the responder’s subjective strategy to indicate that an adjective had previously been seen or not. Further, median reaction times (RTs) were calculated from correctly recalled target adjectives (i.e., a confidence rating above three during the recall phase) for each participant. To better approximate a normal distribution, trial-level RTs were modelled on the log scale. Please see Table S3.1 in the supplementary information for descriptive statistics of all self-referential memory measures.

Autistic traits have been suggested to moderate the self-reference effect (Lombardo et al., 2007; Toichi et al., 2002). To control for autistic traits, we used the Autism Quotient (AQ) questionnaire (Baron-Cohen et al., 2001), which was completed before the learning phase of the self-referential memory task. There were no significant differences between adolescents and adults in the current study on the AQ (*t*(95) = -1.19, *p* = .24; see Table S7 in the supplementary information) and all results held after controlling for autistic traits (see our supplementary information Tables XA to XG).

#### Director Task

To assess age-related changes in the Director task, filler trials (not designed to test participants perspective taking abilities) were excluded from the analyses (Dumontheil et al., 2010). Due to a task specific technical error, perspective taking accuracy data from two adolescent participants could not be analyzed, leaving a total sample size of *N* = 95 for the analyses of perspective taking accuracy. First, accuracy was modelled at the trial level using the binominal distribution (i.e., logistic regression). For each trial type (experimental and control), accuracy measures were obtained by coding correct responses as “1s” and incorrect responses as “0s”. Second, median RTs were calculated from correct trials (experimental and control). Please see Table S3.2 in the supplementary information for descriptive statistics of all perspective taking measures.

#### Relationship between Self-Referential Memory and Perspective Taking

Only participants with complete data sets (i.e., self-referential memory task data and Director task data) were included in the analyses of the relation between self-referential memory and perspective taking, leaving a total sample size of *N* = 93. For those analyses, we computed self-referential difference scores for each participant by subtracting self-referential performance measures (i.e., memory sensitivity and response bias) for self-related target adjectives from performance measures for town-related target adjectives. Larger self-referential difference scores indicate a stronger bias towards self-referentially encoded adjectives as compared to town-referentially encoded adjectives.

#### Age-Related Analysis

For each dependent variable we assessed any association with age, the independent variable of interest. This was used as a continuous variable and standardized. We started each analysis by assessing whether, relative to the linear trend of age alone, the quadratic and cubic trends of age provided a better fit to the data (Luna et al., 2004). Polynomials were orthogonalized to avoid multicollinearity. Afterwards, the model with the lowest AIC value – which indicates better model fit – was selected (Akaike, 1974). Model fits were further compared with likelihood ratio tests. If the difference was not statistically significant (*p* < .05), the more parsimonious model was retained (see Table S5 in the supplementary information for a summary of all model specifications and Table S6 for a summary of model fit indices). Across both comparison methods, the best fitting model always involved only the linear function of age. Moreover, a condition term (self vs. town) was used in the self-referential memory task for memory sensitivity and response bias, while a trial type term (experimental vs. control) was used in the Director task. Self-referential memory reaction times analyses also included a category term (target vs. distractor). Each of these terms were allowed to interact with one another. All data was modelled using mixed-effect models (“lme4” package version 1.1-21; Bates et al., 2018) in R version 3.6.3 (R Core Team, 2016). The resulting coefficients are unstandardized. Subject-level random intercepts were included for all models (Baayen et al., 2008). To determine the random effects structures of our mixed-effects models, we began with the maximal model in order to minimize Type I error (Barr, 2013). When the maximal model gave convergence errors, we removed correlations between random slopes and random intercepts, and, finally, removed random slopes for interaction effects. The resulting random effect structures are available in the supplementary information (Table S5). Main effects and interactions of the best fitting models were inspected using omnibus Type III *F* tests with Satterthwaite approximations for degrees of freedom for linear models and Wald *X^2^* tests for generalized models. Significant main effects and interactions were further inspected with planned comparisons and Bonferroni-corrected post-hoc comparisons using the emmeans package version 1.4.4 (Lenth & Lenth, 2018). The same package was used to convert *F* values of significant main effects and interactions to estimated effect sizes of η_p_^2^ (partial eta-squared; confidence interval = 95%). For full details about the fixed- and random-effects structure of all models see “Full Model Specification and Results” in the supplementary information. Also, see Table S4 in the supplementary information for descriptive statistics and correlations between age, perspective taking accuracy and self-referential difference scores.

## Results

### Self-Referential Memory

#### Memory Sensitivity

**Figure 3:**
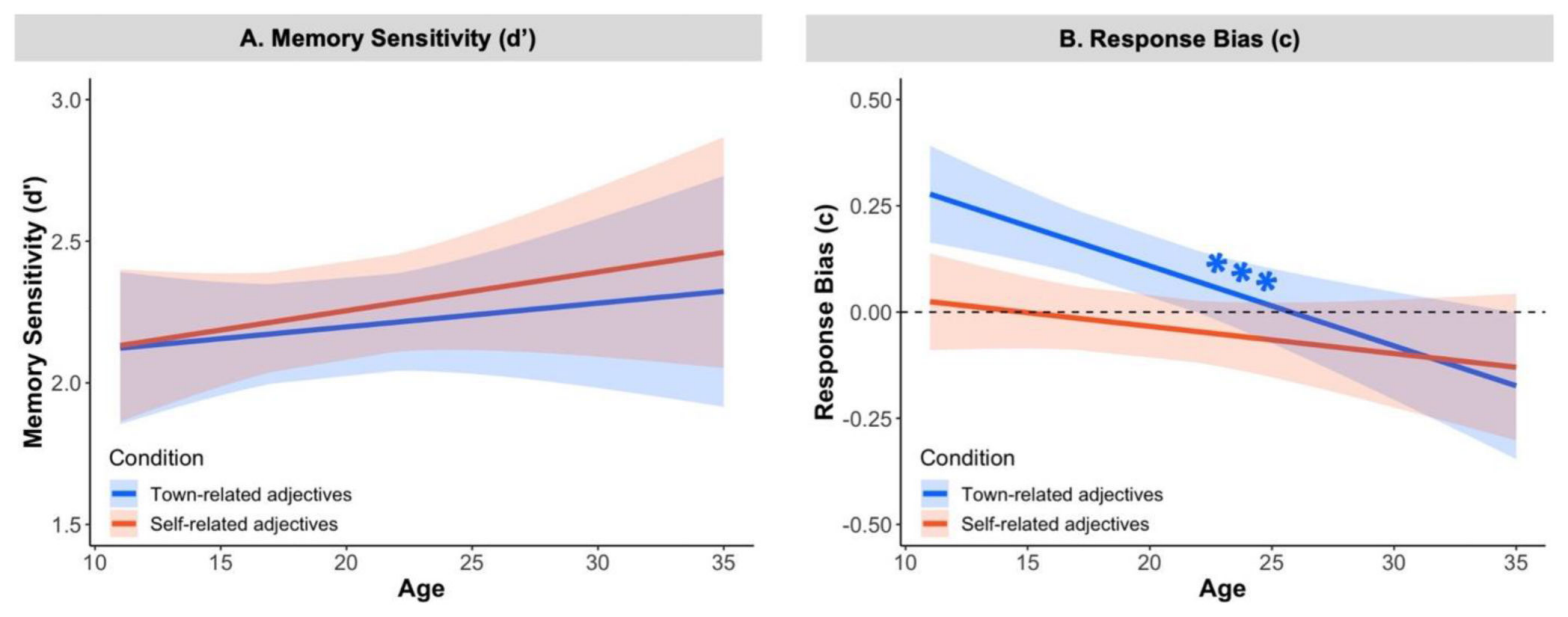
Effect of Age on Memory Sensitivity and Response Bias in the Self-Referential Memory Task *Note*. (**A**) Effect of age on memory sensitivity. Mean *d’* (y-axis) is plotted as a function of age (x-axis) for each condition. There was no significant change with age in the efficiency of processing self (vs. town) related adjectives. (**B**) Effect of age on response bias. Mean *c* (y-axis) is plotted as a function of age (x-axis) for each condition. With increasing age, participants demonstrated a reduction in “conservative” response bias for town-related adjectives, which indicates that participants became more likely to report town-related adjectives as present during the learning phase (slope_town_ = -0.02, SE = .005, *p*_Bonf_ > .001). All colored lines and shaded .95 confidence intervals (CIs of the fixed effects) show the linear trends as estimated by the linear mixed-effects models (A & B). *** *p*_Bonf_ < .001.

First, we examined the relation between age and memory sensitivity (i.e., the ability to discriminate between target adjectives and distractor adjectives) in the self-referential memory task (see Figure 3A below; hypothesis 1). To examine how participants’ memory sensitivity (*d’*) changed as a function of age (continuous) and condition (self vs. town), we ran a linear mixed-effects model. Contrary to our predictions, we did not find any main effects or interactions (*p*s > .32; see Model A and Table A1-2 in the supplementary information). This suggests that we did not observe a significant egocentric memory bias towards self-related adjectives, nor did we observe that memory sensitivity for self- and town-related adjectives was significantly modulated by age in our sample.

#### Memory Response Bias

To leverage the full signal detection theory framework (Anderson, 2015), we also examined the relation between age and response bias (i.e., the responder’s subjective decision strategy to indicate whether an adjective had previously been seen or not) in the self-referential memory task. An exploratory linear mixed-effects model on response biases (*c*) revealed a small Age x Condition interaction effect, χ^2^ (1) = 4.73, *p* = .03, η_p_^2^ = .05 (see Figure 3B below as well as Model B and Table B in the supplementary information). The magnitude of a conservative response bias decreased with age only for town-related adjectives (slope_town_ = -0.02, SE = .005, *p*_Bonf_ > .001), and not for self-related adjectives (*p*_Bonf_ > .22). This finding indicates that participants of all ages used a similar decision criterion when recalling self-related adjectives. In contrast, compared to older participants, younger participants were more likely to use a conservative decision criterion when recalling town-related adjectives. That is, younger participants were more likely to respond that town-related adjectives had not previously been presented during the learning phase, independently of whether or not they had actually been seen.

#### Reaction Times

To examine how participants’ reaction times changed as a function of age (continuous), condition (self vs. town), and category (target vs. distractor), we ran a linear mixed-effects model. This model revealed a medium to large Age x Condition x Category interaction, χ^2^ (1) = 14.10, *p* < .001, η_p_^2^ = .13 (also see Figure S1, Model C, and Table C in the supplementary information; hypothesis 1). Post-hoc contrasts suggested that this was driven by RTs for town-related target adjectives becoming overall faster with age compared to RTs for self-related target adjectives, contrast_town(target) – self(target)_ = -0.01, SE = .002, *p* = .02. In contrast, RTs for town-related distractor adjectives became overall slower with increasing age compared to RTs for self-related distractor adjectives, contrast_town(distractor) – self(distractor)_ = 0.01, SE = .002, *p* = .001.

### Perspective Taking

#### Accuracy

**Figure 4:**
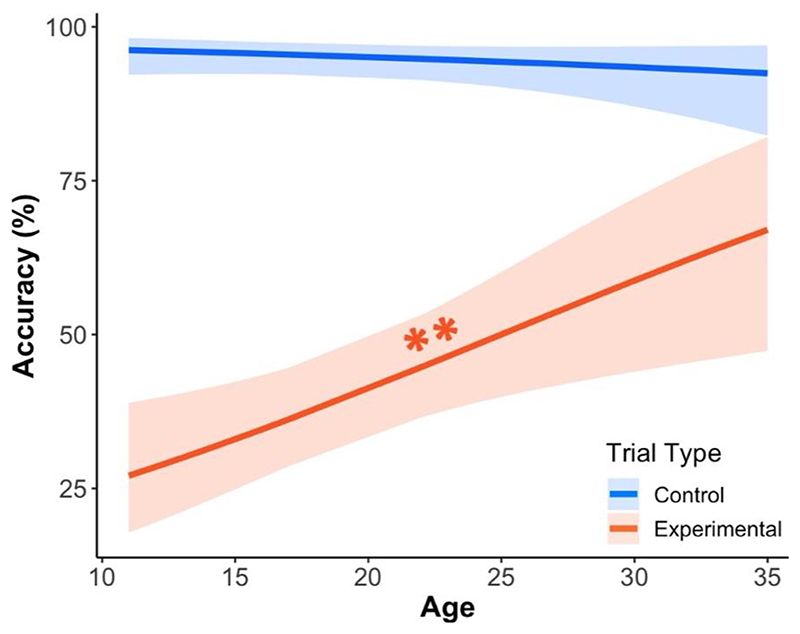
Effect of Age on Perspective Taking Accuracy in the Director Task *Note*. Mean percentage correct (y-axis) is plotted as a function of age (x-axis) for each trial type. During experimental trials, participants had to consider the director’s perspective to select the correct answer, while this was not necessary to correctly answer in control trials. With increasing age, participants demonstrated a greater ability to take account of another person’s perspective (slope _experimental_ = 0.07, SE = .02, *p*_Bonf_ = .01). All colored lines and shaded .95 CIs show the linear trends as estimated by the trial-level generalized linear mixed-effects model. ** *p*_Bonf_ < .01.

The generalized linear mixed-effects model on perspective taking accuracy in the Director task showed a large significant main effect of the experimental treatment (χ^2^ (1) = 122.84, *p* < .001, η_p_^2^ = .67; see Figure 4 below as well as Model D and Table D in the supplementary information; hypothesis 2). Planned contrasts showed that this was due to lower accuracy in the experimental condition relative to the control condition (contrast _control – experimental_ = 3.35, SE = .30, *p* < .001). In addition, this effect was modulated by a small Age x Trial type interaction effect, χ^2^ (1) = 8.54, *p* = .003, η_p_^2^ = .08. In line with our predictions, post-hoc analysis suggested that perspective taking accuracy in experimental trials increased with age (slope _experimental_ = 0.07, SE = .02, *p*_Bonf_ = .01), while this was not the case in control trials (*p*_Bonf_ = .55). Thus, younger participants were less likely than older participants to account for the Director’s perspective when making decisions.

#### Reaction Times

To examine how RTs in the Director task changed as a function of age and trial type, we ran a linear mixed-effects model (see Model E and Table E in the supplementary information; hypothesis 2). We observed no main effect of Age, χ^2^ (1) = .57, *p* = .45, a small significant main effect of Trial type whereby RTs in the experimental condition were faster than in the control condition χ^2^ (1) = 21.83, *p* < .001, η_p_^2^ = .19, and no significant Age x Trial type interaction, χ^2^ (1) = .04, *p* = .84.

#### Interrelation between Self-Referential Memory and Perspective Taking

After demonstrating age-related changes in both self-referential memory and perspective taking, we turned to another central question of interest: do participants across our age range show a link between their ability to process self-related adjectives and their ability to infer other people’s perspectives (hypothesis 3)? To address this question, we ran exploratory linear regression analyses to investigate how age and perspective taking accuracy (i.e., mean percentage errors on experimental trials in the Director task) related to self-referential difference scores (memory sensitivity difference scores and response bias difference scores). Both linear regressions showed no significant main or interaction effects (see Models F-G, Tables F-G in the supplementary information), indicating that age-related differences in self-referential memory were unrelated to differences in perspective taking. Furthermore, to examine how participants’ memory sensitivity (*d’*) and response bias (*c*) changed as a function of condition (self vs. town) and perspective taking accuracy, we ran two exploratory linear mixed-effect models. In line with our linear regression findings, both models did not reveal a significant Condition x Accuracy interaction (*p* > .35). Please refer to Table H.1 and Table H.2 in the supplementary information for the model output. Also, Table S4 in the supplementary information summarizes descriptive statistics and correlations for age, perspective taking accuracy, and self-referential difference scores.

#### Summary

We examined three hypotheses. First, we investigated self-referential memory across adolescence and adulthood. Contrary to our predictions, we observed no significant differences in memory sensitivity for self- vs. town-related adjectives, nor did we observe any age effects in our sample, or interactions between age and self- vs. town-related memory (hypothesis 1). However, an exploratory analysis revealed that adolescents were more likely than adults to display a conservative response bias against town-related adjectives but not self-related adjectives. A similar interaction between age and condition (self vs. town) was observed in RTs, suggesting that the response bias was accompanied by faster RTs for town- vs. self-related adjectives during adolescence. Second, we replicated previous Director task findings by showing that younger participants made more egocentric errors in perspective taking than did older participants (hypothesis 2). Participants of all ages took less time to respond correctly to experimental trials relative to control trials, but this condition effect was not modulated by age. Third, we investigated the relationship between self-referential memory and perspective taking abilities but did not observe any association (hypothesis 3).

## Discussion

In the current study, we found age-related changes in self-referential memory and perspective taking during male adolescence. With regard to self-referential memory, we observed no association between age and memory sensitivity for self- and town-related adjectives (hypothesis 1). However, we found age-related changes in memory response biases, which were weaker for self- than town-related adjectives. This was driven by a decrease in memory biases for town-related adjectives, in that young adolescents used a more conservative decision strategy when recalling town-related adjectives, compared to self-related adjectives. Second, we found a continued age-related improvement in perspective taking abilities between adolescence and early adulthood (hypothesis 2). Finally, measures of the memory domain of self-referential processing (memory sensitivity and response bias) were not significantly related to perspective taking (hypothesis 3). Overall, our findings in male adolescents and young adults show an age-related decrease in memory biases for town- compared with self-related adjectives as well as an age-related decrease in egocentric biases in perspective taking. We found no evidence that these two processes are related.

The self-referential memory effect has been well replicated and is thought to be robust (D’Argembeau et al., 2007; Gutchess et al., 2007; Mitchell et al., 2006; Symons & Johnson, 1997). It is therefore surprising that, in the current study, participants did not show a difference in memory sensitivity between self- and town-related adjectives. We speculate that no egocentric bias was observed because we employed a reduced number of stimuli (target adjectives), which was a consequence of time constraints associated with testing in school settings. Previous studies, for example Dégeilh et al. (2015), used a greater number of stimuli (target adjectives) and observed greater memory for self-related items. We do not expect our sample size to have impacted the self-reference effect, given that previous studies with similar (e.g., Cunningham et al., 2014) or smaller samples (D’Argembeau et al., 2007) reported robust effects.

Contrary to our predictions, adolescents and adults showed no significant differences in memory sensitivity (i.e., the difference between the proportion of hits and false alarms) for self- vs. town-related information in the self-referential memory task. However, exploratory analyses revealed age-related differences in response biases (i.e., the magnitude of a conservative response bias decreased with age only for town-related adjectives). The discrepancy between the subjective confidence in the accuracy of the memory (i.e., response bias) and the actual memory performance (i.e., memory sensitivity) could be explained by the differential effect of emotions on both types of functioning. Several studies have shown that emotional valence of stimuli impact more on the subjective sense of recollection than on objective memory performance (Dougal & Rotello, 2007; Phelps & Sharot, 2008; Rotello & Macmillan, 2007; Sharot et al., 2004; Talarico & Rubin, 2003). In other words, even though emotional stimuli intensify the (subjective) recollective experience, response biases may not be a reliable indicator of the (objective) accurate recollection of details. Further support for this argument comes from neuroimaging studies, which have found that the quality of a memory, and the confidence with which a memory is held, rely on dissociable neural mechanisms (see Eichenbaum et al., 2007 for a review; Sharot et al., 2004). For example, Sharot and colleagues (2004) found that, in adults (aged 20-35 years), recalling “neutral” stimuli was related to enhanced activity in the parahippocampal cortex, whereas recalling “emotional” stimuli was associated with enhanced activity in the amygdala. Our results align with this notion, namely, that confidence in one’s memory and actual memory performance might be subserved by partly distinct cognitive processes. Interestingly, the conservative response bias against town-related words was accompanied by reduced reaction times specifically for this category of words. Speculatively, this suggests that adolescents’ tendency to deny remembering town-related words might involve a less effortful recall strategy for town- vs. self-related stimuli.

During adolescence, feedback from the social environment is a valuable and salient source of information (Blakemore & Mills, 2014) used to gain knowledge about the self (Harter, 2012; Moses-Payne et al., 2022; Sebastian et al., 2008; Van der Aar et al., 2018). We speculate that the memory-related findings could be driven by age-related differences in the emotional salience of self-related information (Blakemore & Mills, 2014), possibly coupled with age-related changes in emotion regulation (Silvers et al., 2012). Previous work in young adults demonstrated that emotionally arousing words (vs. less arousing words) are associated with a more liberal response bias (Dougal & Rotello, 2007), thus favoring self-related adjectives. However, in adolescents, heightened emotional arousal of presented adjectives could also result in a conservative bias against less arousing words. For example, given that adolescents are poorer at regulating their emotions in the presence of emotionally salient stimuli (Silvers et al., 2012), they might be less efficient at dividing their attention between self- (more emotionally salient) and town-related (less emotionally salient) information, resulting in prioritizing self-over town-related information. It remains unclear why age should have modulated a conservative bias against town-related adjectives, rather than a liberal bias towards self-related adjectives. We speculate that self-related information might act as a potential “social-emotional” disruptor, by reorienting attention away from information not related to the self. In line with this interpretation, a neuroimaging study by Magis-Weinberg et al. (2017) found heightened right anterior insula activity, a brain region involved in self-regulatory and reward seeking behaviors, during non-social compared to social relational reasoning in adolescents, relative to adults. Furthermore, a behavioral study by Andrews et al. (2019) showed greater self-reported enjoyment of social compared to non-social photographs in adolescents, relative to adults. Conversely, adults self-reported greater enjoyment of non-social than social photographs and chose to spend more time looking at non-social (compared with social) photographs, suggesting that the value of non-social relative to social stimuli increases with age. To test our social-emotional disruptor hypothesis, future studies should investigate whether young adolescents continue to display a conservative bias against town-related adjectives once such potential social-emotional disruptors are removed (e.g., by omitting self-related adjectives).

The results from the Director task in the current sample of male participants replicate previous findings from female samples demonstrating age-related differences in perspective taking abilities (Dumontheil et al., 2010; Dumontheil et al., 2012). In our male sample, we found age-related increases in task accuracy in experimental trials (i.e., trials requiring participants to use information about the director’s perspective) (hypothesis 2). Hence, our results show that the ability to account for someone else’s perspective in order to guide behavior continues to improve throughout adolescence: not only in females, but also in males. Whilst age-related changes in reaction times were not a key measure of interest in the Director task (Humphrey & Dumontheil, 2016), we found that, regardless of age, participants responded more quickly during experimental (vs. control) trials. This is in line with previous findings by Tamnes et al. (2018). We observed no evidence of age-related differences in reaction times. This suggests that age-related improvements in perspective taking are not the result of adults taking more time to respond than adolescents (e.g., adults favoring accuracy over speed).

Together, our findings demonstrate a linear age-related decrease in memory biases for town-related adjectives compared to self-related adjectives, as well as an age-related increase in perspective taking accuracy. However, these tendencies do not seem to be driven by the same processes, insofar as they are not significantly correlated (hypothesis 3). Previous studies have suggested that self- and other-oriented thinking are intertwined deveopmental processes (Cooley, 1983; Crone & Fuligni, 2020) and that self-knowledge can be used to inform others’ mental states and perspectives (Tamir & Mitchell, 2010). Furthermore, these claims were partially supported by studies investigating this relationship in autistic individuals (Lombardo et al., 2007; Toichi et al., 2002). However, the current results indicate no relationship between self-referential memory and perspective taking in typically developing adolescents and young adults. It is possible that memory biases and egocentric biases in perspective taking, as measured here, rely on distinct processes. For example, they might differentially involve executive functions. In line with this, previous work by (Cunningham et al., 2014) showed that executive abilities were not related to an individual’s tendency to preferentially recall self-related information, whereas executive abilities were found to interact with perspective taking in the Director task (Dumontheil et al., 2010).

### Limitations

The cross-sectional findings reported here should be considered in the context of certain limitations. First, we cannot draw any conclusions about causality from this study. Several hypothetical causal mechanisms could underlie the co-development of perspective taking abilities and the self-reference effect. For example, the development of categorical self-concept during adolescence could simultaneously modulate self-referential memory and perspective taking. On the other hand, developing perspective taking abilities could support the formation of a more differentiated self-concept. Alternatively, or in addition, a third construct could causally influence both self-referential memory and perspective taking during development. Future studies are required to disentangle potential causal pathways. Second, we introduced a modified paradigm to investigate self-referential memory by presenting participants with positively valenced adjectives in reference to themselves (social information) and London (non-social information). Previous work suggests that self-reference effects are larger for positively valenced words (Moses-Payne et al., 2022). However, given that we assessed only one domain of social processing (the self-referential domain), it is unclear whether the observed age-related changes in response bias are specific to self-related information or apply to social information (of both positive and negative valence) more generally. Furthermore, the goal of the current study was to assess age-related changes related to the categorical self-concept. Hence, different developmental trajectories may be revealed by parsing other aspects of the self (e.g., agentic self or bodily self). While this was beyond the scope of the current study, future work could examine how the current findings related to distinct facets of the self. Lastly, we recruited exclusively male participants to test for within-group variation, removing variance that could be accounted for by gender differences in perspective taking development during adolescence (Keulers et al., 2010; Maccoby, 1998; Van der Graaff et al., 2014). Future (ideally longitudinal) self-referential memory studies should continue to leverage the signal detection theory framework, use a larger number of positively and negatively valenced social (e.g., self and other) and non-social stimuli (e.g., town), investigate different self-concept domains, and recruit across genders.

### Conclusion

The current findings show that the impact of biases in self-referential memory and perspective taking decreases with age between adolescence and early adulthood. Adolescents were less prone to recognize previously seen town-related adjectives, compared to self-related adjectives. However, this age-related decrease in recognition biases was unrelated to participants’ ability to correctly recall previously seen adjectives (signal) and avoid false recall of new adjectives (noise). In parallel, we found age-related decreases in egocentric biases in perspective taking from adolescence to early adulthood (i.e., perspective taking abilities improved with age). However, these two processes were not related to each other, and therefore might be mediated by different cognitive processes.

## Supplementary Material

Supplementary Information

## Data Availability

Code to reproduce all analyses in the manuscript will be made available on the Open Science Framework https://osf.io/8hf9t/?view_only=048b3543adb942b08490d25c856abc08 and will be live upon publication. Please note that we do not have ethical permission to share participant data.

## Highlights

- Age-related decrease in recognition biases for town vs. self-related adjectives.
- Age-related decrease in egocentric biases in perspective taking.
- Biases in self-referential memory and perspective taking were not related.

## Footnotes

1 We acknowledge ongoing disagreements among professionals (including researchers) and people with autism around the most appropriate and preferred language use when referring to autistic individuals. In this manuscript, we use identity-first language as the latest findings suggest that this formulation is the most common terminology preferred by the autistic community (Taboas, Doepke, & Zimmerman, 2022).

## References

[R1] Akaike H (1974). A new look at the statistical model identification. IEEE Transactions on Automatic Control.

[R2] Anderson ND (2015). Teaching signal detection theory with pseudoscience. Frontiers in Psychology.

[R3] Anderson NH (1968). Likableness ratings of 555 personality-trait words. Journal of Personality and Social Psychology.

[R4] Anwyl-Irvine AL, Massonnié J, Flitton A, Kirkham N, Evershed JK (2020). Gorilla in our midst: An online behavioral experiment builder. Behavior Research Methods.

[R5] Baayen RH, Davidson DJ, Bates DM (2008). Mixed-effects modeling with crossed random effects for subjects and items. Journal of Memory and Language.

[R6] Baron-Cohen S, Hoekstra RA, Knickmeyer R, Wheelwright S (2006). The Autism-Spectrum Quotient (AQ)—Adolescent Version. Journal of Autism and Developmental Disorders.

[R7] Baron-Cohen S, Leslie AM, Frith U (1985). Does the autistic child have a “theory of mind” ?. Cognition.

[R8] Baron-Cohen S, Wheelwright S, Skinner R, Martin J, Clubley E (2001). The autism spectrum quotient (AQ): Evidence from asperger syndrome/high-functioning autism, males and females, scientists and mathematicians. Journal of Autism and Developmental Disorders.

[R9] Barr DJ (2013). Random effects structure for testing interactions in linear mixed-effects models. Frontiers in Psychology.

[R10] Bates D, Kliegl R, Vasishth S, Baayen H (2018). Parsimonious mixed models. ArXiv:150604967 [Stat].

[R11] Blakemore SJ, Mills KL (2014). Is adolescence a sensitive period for sociocultural processing?. Annual Review of Psychology.

[R12] Cole DA, Maxwell SE, Martin JM, Peeke LG, Seroczynski AD, Tram JM, Hoffman KB, Ruiz MD, Jacquez F, Maschman T (2001). The development of multiple domains of child and adolescent self-concept: A cohort sequential longitudinal design. Child Development.

[R13] Cooley CH, O’Brien J (1983). The production of reality: Essays and readings on social interaction.

[R14] Craik FIM, Moroz TM, Moscovitch M, Stuss DT, Winocur G, Tulving E, Kapur S (1999). In search of the self: a positron emission tomography study. Psychological Science.

[R15] Crone EA, Fuligni AJ (2020). Self and others in adolescence. Annual Review of Psychology.

[R16] Cunningham SJ, Brebner JL, Quinn F, Turk DJ (2014). The self-reference effect on memory in early childhood. Child Development.

[R17] Dahl RE (2004). Adolescent brain development: a period of vulnerabilities and opportunities. Keynote address Annals of the New York Academy of Sciences.

[R18] D’Argembeau A, Ruby P, Collette F, Degueldre C, Balteau E, Luxen A, Maquet P, Salmon E (2007). Distinct regions of the medial prefrontal cortex are associated with self-referential processing and perspective taking. Journal of Cognitive Neuroscience.

[R19] Dégeilh F, Guillery-Girard B, Dayan J, Gaubert M, Chételat G, Egler P-J, Baleyte J-M, Eustache F, Viard A (2015). Neural correlates of self and its interaction with memory in healthy adolescents. Child Development.

[R20] Dinulescu S, Alvi T, Rosenfield D, Sunahara CS, Lee J, Tabak BA (2021). Self-referential processing predicts social cognitive ability. Social Psychological and Personality Science.

[R21] Dougal S, Rotello CM (2007). “Remembering” emotional words is based on response bias, not recollection. Psychonomic Bulletin Review.

[R22] Dumontheil I, Apperly IA, Blakemore S-J (2010). Online usage of theory of mind continues to develop in late adolescence. Developmental Science.

[R23] Dumontheil I, Hillebrandt H, Apperly IA, Blakemore S-J (2012). Developmental differences in the control of action selection by social information. Journal of Cognitive Neuroscience.

[R24] Eichenbaum H, Yonelinas AP, Ranganath C (2007). The medial temporal lobe and recognition memory. Annual Review of Neuroscience.

[R25] Erikson E (1968). Identity: Youth in crisis.

[R26] Forsberg A, Blume CL, Cowan N (2021). The development of metacognitive accuracy in working memory across childhood. Developmental Psychology.

[R27] Frith U, Frith CD (2003). Development and neurophysiology of mentalizing. Philosophical Transactions of the Royal Society of London Series B: Biological Sciences.

[R28] Gallagher HL, Frith CD (2003). Functional imaging of ‘theory of mind’. Trends in Cognitive Sciences.

[R29] Gutchess AH, Kensinger EA, Yoon C, Schacter DL (2007). Ageing and the self-reference effect in memory. Memory.

[R30] Halpin JA, Puff CR, Mason HF, Marston SP (1984). Self-reference encoding and incidental recall by children. Bulletin of the Psychonomic Society.

[R31] Harter S (1990). Developmental differences in the nature of self-representations: Implications for the understanding, assessment, and treatment of maladaptive behavior. Cognitive Therapy and Research.

[R32] Harter S, Leary MR, Tangney JP (2012). Handbook of self and identity.

[R33] Henderson HA, Zahka NE, Kojkowski NM, Inge AP, Schwartz CB, Hileman CM, Coman DC, Mundy PC (2009). Self-referenced memory, social cognition, and symptom presentation in autism. Journal of Child Psychology and Psychiatry.

[R34] Humphrey G, Dumontheil I (2016). Development of risk-taking, perspective-taking, and inhibitory control during adolescence. Developmental Neuropsychology.

[R35] Keulers EH, Evers EA, Stiers P, Jolles J (2010). Age, sex, and pubertal phase influence mentalizing about emotions and actions in adolescents. Developmental Neuropsychology.

[R36] Kelley WM, Macrae CN, Wyland CL, Caglar S, Inati S, Heatherton TF (2002). Finding the self? An event-related fMRI study. Journal of Cognitive Neuroscience.

[R37] Klein SB, Kihlstrom JF (1986). Elaboration, organization, and the self-reference effect in memory. Journal of Experimental Psychology: General.

[R38] Lenth R, Lenth MR (2018). Package ‘lsmeans’. The American Statistician.

[R39] Locke SM, Robinson OJ (2021). Affective bias through the lens of Signal Detection Theory. Computational Psychiatry.

[R40] Lombardo MV, Barnes JL, Wheelwright SJ, Baron-Cohen S (2007). Self-referential cognition and empathy in autism. PLoS ONE.

[R41] Luna B, Garver KE, Urban TA, Lazar NA, Sweeney JA (2004). Maturation of cognitive processes from late childhood to adulthood. Child Development.

[R42] Maccoby EE (1998). The two sexes: Growing up apart, coming together.

[R43] Magis-Weinberg L, Blakemore SJ, Dumontheil I (2017). Social and nonsocial relational reasoning in adolescence and adulthood. Journal of Cognitive Neuroscience.

[R44] Mitchell JP, Macrae CN, Banaji MR (2006). Dissociable medial prefrontal contributions to judgments of similar and dissimilar others. Neuron.

[R45] Montemayor R, Eisen M (1975). Paper presented at the Biennial Meeting of the Society for Research in Child Development.

[R46] Moses-Payne ME, Chierchia G, Blakemore SJ (2022). Age-related changes in the impact of valence on self-referential processing in female adolescents and young adults. Cognitive Development.

[R47] Phelps EA, Sharot T (2008). How (and why) emotion enhances the subjective sense of recollection. Current Directions in Psychological Science.

[R48] Psychology Software Tools, Inc Psychology Software Tools (Version 3.0) [E-Prime].

[R49] R Core Team (2016). R: A language and environment for statistical computing. R Foundation for Statistical Computing.

[R50] Ray RD, Shelton AL, Hollon NG, Michel BD, Frankel CB, Gross JJ, Gabrieli JD (2009). Cognitive and neural development of individuated self-representation in children. Child Development.

[R51] Renner CH, Renner MJ (2001). But I thought I knew that: Using confidence estimation as a debiasing technique to improve classroom performance. Applied Cognitive Psychology: The Official Journal of the Society for Applied Research in Memory and Cognition.

[R52] Rogers TB, Kuiper NA, Kirker WS (1977). Self-reference and the encoding of personal information. Journal of Personality and Social Psychology.

[R53] Rosenstreich E, Ruderman L (2016). Not sensitive, yet less biased: A signal detection theory perspective on mindfulness, attention, and recognition memory. Consciousness and Cognition.

[R54] Rotello CM, Macmillan NA (2007). Psychology of Learning and Motivation.

[R55] Sawyer SM, Azzopardi PS, Wickremarathne D, Patton GC (2018). The age of adolescence. The Lancet Child Adolescent Health.

[R56] Sebastian C, Burnett S, Blakemore S-J (2008). Development of the self-concept during adolescence. Trends in Cognitive Sciences.

[R57] Sharot T, Delgado MR, Phelps EA (2004). How emotion enhances the feeling of remembering. Nature Neuroscience.

[R58] Silvers JA, McRae K, Gabrieli JDE, Gross JJ, Remy KA, Ochsner KN (2012). Age-related differences in emotional reactivity, regulation, and rejection sensitivity in adolescence. Emotion.

[R59] Spreng RN, Mar RA, Kim AS (2009). The common neural basis of autobiographical memory, prospection, navigation, theory of mind, and the default mode: a quantitative meta-analysis. Journal of Cognitive Neuroscience.

[R60] Stanislaw H, Todorov N (1999). Calculation of signal detection theory measures. Behavior Research Methods, Instruments, Computers.

[R61] Sui J, Zhu Y (2005). Five-year-olds can show the self-reference advantage. International Journal of Behavioral Development.

[R62] Sweatman H, Lawrence R, Chai XJ (2022). Development of self-referential effect on memory recollection. Child Development.

[R63] Symeonidou I, Dumontheil I, Chow W-Y, Breheny R (2016). Development of online use of theory of mind during adolescence: An eye-tracking study. Journal of Experimental Child Psychology.

[R64] Symons CS, Johnson BT (1997). The self-reference effect in memory: a meta-analysis. Psychological Bulletin.

[R65] Taboas A, Doepke K, Zimmerman C (2022). Preferences for identity-first versus person-first language in a US sample of autism stakeholders. Autism.

[R66] Talarico JM, Rubin DC (2003). Confidence, not consistency, characterizes flashbulb memories. Psychological Science.

[R67] Tamir DI, Mitchell JP (2010). Neural correlates of anchoring-and-adjustment during mentalizing. Proceedings of the National Academy of Sciences.

[R68] Tamnes CK, Overbye K, Ferschmann L, Fjell AM, Walhovd KB, Blakemore SJ, Dumontheil I (2018). Social perspective taking is associated with self-reported prosocial behavior and regional cortical thickness across adolescence. Developmental Psychology.

[R69] Todd AR, Hanko K, Galinsky AD, Mussweiler T (2011). When focusing on differences leads to similar perspectives. Psychological Science.

[R70] Toichi M, Kamio Y, Okada T, Sakihama M, Youngstrom EA, Findling RL, Yamamoto K (2002). A lack of self-consciousness in autism. American Journal of Psychiatry.

[R71] Van der Aar LPE, Peters S, Crone EA (2018). The development of self-views across adolescence: Investigating self-descriptions with and without social comparison using a novel experimental paradigm. Cognitive Development.

[R72] Van der Graaff J, Branje S, De Wied M, Hawk S, Van Lier P, Meeus W (2014). Perspective taking and empathic concern in adolescence: gender differences in developmental changes. Developmental Psychology.

[R73] Wylie GR, Yao B, Sandry J, DeLuca J (2021). Using signal detection theory to better understand cognitive fatigue. Frontiers in Psychology.

