# Supplementary material for: Perspective Taking and Memory for Self- and Town-Related Information in Male Adolescents and Young Adults
Source: Cogn Dev. Author manuscript; Available in PMC 2023 Nov 6. (PMC7615281; doi:10.1016/j.cogdev.2023.101356)
Supplement: Supplementary Information [file EMS190285-supplement-Supplementary_Information.docx]

**Supplementary Information**

**Table S1**

*Participant Information*

|  | **Adolescents**  (*n* = 50; aged 11.2-17.5) | | | |  | **Adults**  (*n* = 47; aged 22.2-35.6) | | | |
| --- | --- | --- | --- | --- | --- | --- | --- | --- | --- |
|  | *M* | *SD* | | Range |  | *M* | *SD* | | Range |
| **Age** (years) | 13.96 | 1.48 | | 11.2-17.5 |  | 26.04 | 3.62 | | 22.2-35.6 |
| **% Race** |  | | | |  |  | | | |
| Asian | 6 | | | |  | 64 | | | |
| Black | 0 | | | |  | 11 | | | |
| White | 82 | | | |  | 19 | | | |
| Mixed | 10 | | | |  | 2 | | | |
| Other | 2 | | | |  | 4 | | | |
| **% Parental highest education attainment** | Mother | | Father | |  | Mother | | Father | |
| O-Level or GCSE  (UK national exams at 16) | 2 | | 6 | |  | 13 | | 17 | |
| AS or A-Level  (UK national exams at 18) | 14 | | 12 | |  | 13 | | 15 | |
| Undergraduate  (e.g., BSc/BA) | 50 | | 40 | |  | 45 | | 28 | |
| Postgraduate  (e.g., MSc/MA, PhD) | 28 | | 36 | |  | 23 | | 21 | |
| N/A | 6 | | 6 | |  | 2 | | 8 | |
| Don’t Know | 0 | | 0 | |  | 4 | | 11 | |

*Note.* Descriptive statistics of participants included in the analyses (all male) by age group, race, and parental highest education attainment. Race and parental highest education attainment were reported by adult participants or the primary caregiver of the adolescent participants. M = Mean, SD = Standard deviation.

**Table S2**

*Target and Distractor Adjectives used in the Self-Referential Memory Task*

| **Self** | |  | **Town** | |
| --- | --- | --- | --- | --- |
| Target(*n* = 20) | Distractor(*n* = 80) |  | Target(*n* = 20) | Distractor(*n* = 80) |
| \| Kind \| \| --- \| \| Arty \| \| Happy \| \| Smart \| \| Eager \| \| Chatty \| \| Joyful \| \| Honest \| \| Healthy \| \| Relaxed \| \| Cautious \| \| Athletic \| \| Unafraid \| \| Practical \| \| Attentive \| \| Emotional \| \| Respectful \| \| Thoughtful \| \| Imaginative \| \| Warm-hearted \| | \| Calm \| Reliable \| \| --- \| --- \| \| Fair \| Sociable \| \| Brave \| Sensible \| \| Funny \| Tolerant \| \| Jolly \| Truthful \| \| Proud \| Trustful \| \| Active \| Talented \| \| Clever \| Pleasant \| \| Caring \| Confident \| \| Giggly \| Sensitive \| \| Humble \| Talkative \| \| Modest \| Interested \| \| Polite \| Optimistic \| \| Sporty \| Supportive \| \| Careful \| Cooperative \| \| Curious \| Hard-working \| \| Helpful \| Intelligent \| \| Patient \| Inquisitive \| \| Cheerful \|  \| \| Creative \|  \| \| Fearless \|  \| \| Generous \|  \| |  | \| Big \| \| --- \| \| Rich \| \| Tidy \| \| Cosy \| \| Local \| \| Foggy \| \| Green \| \| Pretty \| \| Remote \| \| Classic \| \| Ancient \| \| Spacious \| \| Friendly \| \| Charming \| \| Traditional \| \| Thrilling \| \| Welcoming \| \| Historical \| \| Adventurous \| \| Multicultural \| | \| Busy \| Friendly \| \| --- \| --- \| \| Cute \| Medieval \| \| Cold \| Pleasant \| \| Grey \| Relaxing \| \| Huge \| Stunning \| \| Safe \| Terrific \| \| Tiny \| Touristy \| \| Loud \| Peaceful \| \| Nice \| Beautiful \| \| Clean \| Colourful \| \| Quiet \| Enjoyable \| \| Rainy \| Energetic \| \| Small \| Fantastic \| \| Magic \| Industrial \| \| Cloudy \| Impressive \| \| Famous \| Fashionable \| \| Modern \| International \| \| Amazing \|  \| \| Crowded \|  \| \| Elegant \|  \| \| Popular \|  \| \| Wealthy \|  \| |

**Table S3.1**

*Self-Referential Memory: Descriptive Statistics*

|  | **Adolescents**  (*n* = 50; aged 11.2-17.5) | | |  | **Adults**  (*n* = 47; aged 22.2-35.6) | | |  |  |  |
| --- | --- | --- | --- | --- | --- | --- | --- | --- | --- | --- |
|  | *M* | *SD* | Range |  | *M* | *SD* | Range |  | *t*-value | *p*-value |
| **Memory sensitivity** (self) | 2.19 | 0.79 | 0.47-4.17 |  | 2.32 | 0.97 | -0.28-3.77 |  | -0.67 | .50 |
| **Memory sensitivity** (town) | 2.15 | 0.58 | 0.55-3.49 |  | 2.24 | 0.82 | 0.13-3.72 |  | -0.63 | .53 |
| **Response bias** (self) | 0.02 | 0.29 | -0.94-0.62 |  | -0.08 | 0.39 | -0.93-0.65 |  | 1.39 | .17 |
| **Response bias** (town) | 0.22 | 0.32 | -0.48-0.76 |  | -0.01 | 0.34 | -0.93-0.81 |  | 3.32 | **.001** |
| **Reaction times** (self, target) | 2543.35 | 1895.69 | 98.81-22161.92 |  | 2400.7 | 1921.47 | 167.80-22104.2 |  | 1.62 | .11 |
| **Reaction times** (self, distractor) | 2779.39 | 2049.79 | 4.51-32989.66 |  | 2500.8 | 2130.48 | 48.40-47663.40 |  | 4.09 | **<.001** |
| **Reaction times** (town, target) | 2631.45 | 1784.78 | 6.75-18845.60 |  | 2262.70 | 1504.52 | 4.20-20692.20 |  | 4.82 | **<.001** |
| **Reaction times** (town, distractor) | 2645.48 | 2028.91 | 9.06-29229.33 |  | 2494.00 | 2030.93 | 165.20-23731.40 |  | 2.29 | **.02** |
| **Memory sensitivity difference scores** (self-town) | 0.04 | 0.68 | -1.48-1.91 |  | 0.07 | 0.65 | -1.21-1.64 |  | -0.22 | .83 |
| **Response bias difference scores** (self-town) | -0.21 | 0.34 | -0.80-0.62 |  | -0.08 | 0.41 | -1.07-1.02 |  | -1.68 | .10 |

*Note.* Descriptive statistics of self-referential memory sensitivity, response bias, and rection times for both self- and town-related adjectives as well as memory sensitivity- and response bias difference scores. Additionally, reaction times are reported for both target and distractor adjectives. Difference scores were computed for each participant by subtracting self-referential performance measures (i.e., memory sensitivity and response bias) for self-related target adjectives from performance measures for town-related target adjectives. Larger self-referential difference scores indicate a stronger bias towards self-referentially encoded adjectives compared to town-referentially encoded adjectives. M = Mean, SD = Standard deviation.

**Table S3.2**

*Perspective Taking: Descriptive Statistics*

|  | **Adolescents**  (*n* = 50; aged 11.2-17.5) | | |  | **Adults**  (*n* = 47; aged 22.2-35.6) | | |  |  |  |
| --- | --- | --- | --- | --- | --- | --- | --- | --- | --- | --- |
|  | *M* | *SD* | Range |  | *M* | *SD* | Range |  | *t*-value | *p*-value |
| **Accuracy** (experimental) | 0.35 | 0.48 | 0-1 |  | 0.50 | 0.51 | 0-1 |  | -3.93 | **<.001** |
| **Accuracy** (control) | 0.95 | 0.23 | 0-1 |  | 0.91 | 0.28 | 0-1 |  | 1.75 | .08 |
| **Reaction times** (experimental) | 2742.00 | 512.48 | 358.00- 3997.00 |  | 2733.00 | 592.75 | 304.00- 3963.00 |  | 0.23 | .82 |
| **Reaction times**  (control) | 2875.00 | 509.82 | 297.00-3981.00 |  | 2855.00 | 601.64 | 326.00-3978.00 |  | 0.49 | .63 |

*Note.* Descriptive statistics of perspective taking accuracy and reaction times for both experimental and control trial types.
M = Mean, SD = Standard deviation.

**Table S4**

*Descriptive Statistics and Correlations between Age, Perspective Taking Accuracy, and Self-Referential Difference Scores*

| Variable | *n* | *M* | *SD* | 1 | 2 | 3 | 4 |
| --- | --- | --- | --- | --- | --- | --- | --- |
| 1. **Age** | 94 | 19.74 | 6.68 | - |  |  |  |
| 2. **Experimental accuracy** | 92 | 0.43 | 0.31 | **0.28^*^** | - |  |  |
| 3. **Memory sensitivity difference scores**  (self-town) | 94 | 0.06 | 0.66 | 0.04 | -0.07 | - |  |
| 4. **Response bias difference scores**  (self-town) | 94 | -0.15 | 0.38 | **0.23^*^** | 0.15 | -0.15 | - |

*Note.* Experimental accuracy represents perspective taking accuracy in experimental trials of the Director task. M = Mean, SD = Standard deviation, ^*^*p*< .05, uncorrected.

**Full Model Specification and Results**

For each model described in the manuscript, we report here its full random-effects structure and fixed effect estimates.

**Table S5**

*Summary of Model Specifications*

|  | **Dependent Variable** | **Fixed Effects** | **Random Effects** |
| --- | --- | --- | --- |
| **Hypothesis 1** |  |  |  |
| Model A | Memory sensitivity (dprime) | Age, Condition | Participants |
| Model B | Response bias (c) | Age, Condition | Participants |
| Model C | RTs (log-transformed) | Age, Condition, Category | Participants |
| **Hypothesis 2** |  |  |  |
| Model D | Perspective taking accuracry | Age, Trial type | Participants, Trial type |
| Model E | Perspective taking RTs | Age, Trial type | Participants |
| **Hypothesis 3** |  |  |  |
| Model F | Memory sensitivity difference scores | Age, Perspective taking accuracy | - |
| Model G | Response bias difference scores | Age, Perspective taking accuracy | - |
| **Exploratory** |  |  |  |
| Model X1.1 | Learning phase ratings | Age, Condition | Participants |
| Model X1.2 | Learning phase RTs (log-transformed) | Age-squared, Condition | Participants |
| Model X2.1 | Recall phase ratings | Age, Condition | Participants |
| Model X2.2 | Recall phase RTs (log-transformed) | Age, Condition | Participants |

*Note.* Models A, B, F, and G have used person level data, whilst the others used trial-level data. Age was used as a continuous variable and standardized. Condition was a binary variable describing whether adjectives in the self-referential memory task were self- or town-related. Trial type was also a binary variable describing whether perspective taking accuracy was measured during an experimental or control trial. Self-referential memory reaction time analyses also included a category term describing whether adjectives were presented during the learning phase (targets) or not (distractors). As fixed effects, we investigated the interaction (as well as all lower-level interactions) between all independent variables. Subject-level random intercepts (participants) were included for all models, and we employed maximal random slopes for the within-subject factor (trial type) in Model D (Barr, 2013). For further model specific information please see the statistical analysis section of the main manuscript. RTs = reaction times.

**Self-Referential Memory (Hypothesis 1)**

***Model A: Memory Sensitivity***

We examined how age and condition (self vs. town) influenced memory sensitivity (and accuracy) with a linear mixed-effects model. All predictors of interest were included in the model as interacting fixed effects. The model also included random intercepts for each participant. In this model and all subsequent models, age was treated as a continuous variable for which we computed orthogonal polynomials to avoid multicollinearity.

**Table A.1**

*Memory Sensitivity: Mixed Model Omnibus Results*

|  | $\chi$^2^ | *df* | *p*-value |
| --- | --- | --- | --- |
| Intercept | 907.28 | 1 | **<.001** |
| Age | 0.98 | 1 | .32 |
| Condition | 0.68 | 1 | .41 |
| Age x Condition | 0.27 | 1 | .60 |

**Table A.2**

*Memory Accuracy (Trial-Level): Mixed Model Omnibus Results*

|  | $\chi$^2^ | *df* | *p*-value |
| --- | --- | --- | --- |
| Intercept | 627.65 | 1 | **<.001** |
| Age | 0.14 | 1 | .71 |
| Condition | 1.29 | 1 | .26 |
| Age x Condition | 0.59 | 1 | .44 |

***Model B: Response Bias***

We examined how age and condition (self vs. town) influenced response bias with a linear mixed-effects model. All predictors of interest were included in the model as interacting fixed effects. The model also included random intercepts for each participant.

**Table B**

*Response Bias: Mixed Model Omnibus Results*

|  | $\chi$^2^ | *df* | *p*-value |
| --- | --- | --- | --- |
| Intercept | 1.99 | 1 | .16 |
| Age | 8.43 | 1 | **.004** |
| Condition | 14.77 | 1 | **<.001** |
| Age x Condition | 4.73 | 1 | **.03** |

***Model C: Memory Reaction Times***

We examined how age, condition (self vs. town), and category (target vs. distractor adjectives) influenced reaction times (RTs) for recognized adjectives with a linear mixed-effects model. RTs were log-transformed to better approximate normal distribution. All predictors of interest were included in the model as interacting fixed effects. The model also included random intercepts for each participant.

**Table C**

*Memory Reaction Times: Mixed Model Omnibus Results*

|  | $\chi$^2^ | *df* | *p*-value |
| --- | --- | --- | --- |
| Intercept | 71462.24 | 1 | **<.001** |
| Age | 2.31 | 2 | .13 |
| Condition | 3.10 | 1 | .08 |
| Category | 12.32 | 1 | **<.001** |
| Age x Condition | 0.003 | 2 | .95 |
| Age x Category | 0.69 | 2 | .41 |
| Condition x Category | 1.90 | 1 | .17 |
| Age x Condition x Category | 14.10 | 2 | **<.001** |

**Figure S1**

*Effect of Age on Memory RTs in the Self-Referential Memory Task*

*Note*. Median RTs (y-axis) are plotted as a function of age (x-axis) for each condition and category. All colored lines and shaded .95 CIs show the linear trends as estimated by the linear mixed-effects models.

**Perspective Taking (Hypothesis 2)**

***Model D: Perspective Taking Accuracy***

We examined how age and trial type (experimental vs. control) influenced perspective taking accuracy with a generalized linear mixed-effects model. All predictors of interest were included in the model as interacting fixed effects. The model also included random intercepts for each participant and allowed for different slopes across trial type.

**Table D**

*Perspective Taking Accuracy: Mixed Model Omnibus Results*

|  | $\chi$^2^ | *df* | *p*-value |
| --- | --- | --- | --- |
| Intercept | 62.52 | 1 | **<.001** |
| Age | 1.03 | 1 | .31 |
| Trial type | 122.84 | 1 | **<.001** |
| Age x Trial type | 8.54 | 1 | **.003** |

***Model E: Perspective Taking Reaction Times***

We examined how age and trial type (experimental vs. control) influenced perspective taking RTs with a linear mixed-effects model. All predictors of interest were included in the model as interacting fixed effects. The model also included random intercepts for each participant.

**Table E**

*Perspective Taking Reaction Times: Mixed Model Omnibus Results*

|  | $\chi$^2^ | *df* | *p*-value |
| --- | --- | --- | --- |
| Intercept | 12170.89 | 1 | **<.001** |
| Age | 0.57 | 1 | .45 |
| Trial type | 21.83 | 1 | **<.001** |
| Age x Trial type | 0.04 | 1 | .84 |

**Interrelation between Self-Referential Memory and Perspective Taking (Hypothesis 3)**

***Model F: Relation between Memory Sensitivity and Perspective Taking Accuracy***

With a linear regression, we examined how age and perspective taking accuracies related to memory sensitivity difference scores.

**Table F**

*Relation between Memory Sensitivity Difference Scores and Perspective Taking Accuracy: Regression Results*

|  | *F* | *df* | *p*-value |
| --- | --- | --- | --- |
| Intercept | 1.54 | 1,88 | .22 |
| Age | 0.28 | 1,88 | .60 |
| Experimental accuracy | 1.04 | 1,88 | .31 |
| Age x Experimental accuracy | 1.00 | 1,88 | .32 |

***Model G: Relation between Response Bias and Perspective Taking Accuracy***

With a linear regression, we examined how age and perspective taking accuracies related to response bias difference scores.

**Table G**

*Relation between Response Bias Difference Scores and Perspective Taking Accuracy: Regression Results*

|  | *F* | *df* | *p*-value |
| --- | --- | --- | --- |
| Intercept | 9.07 | 1,88 | **.003** |
| Age | 1.89 | 1,88 | .17 |
| Experimental accuracy | 0.92 | 1,88 | .34 |
| Age x Experimental accuracy | 0.16 | 1,88 | .69 |

**Exploratory Analyses: Interrelation between Self-Referential Memory and Perspective Taking**

To examine how participants’ memory sensitivity (*d’*) and response bias (*c*) changed as a function of condition (self vs. town) and perspective taking accuracy, we ran two linear mixed-effect models. Both did not reveal a significant Condition x Accuracy interaction (*p*> .35).

**Table H.1**

*Relation between Memory Sensitivity and Perspective Taking Accuracy: Mixed Model Omnibus Results*

|  | $\chi$^2^ | *df* | *p*-value |
| --- | --- | --- | --- |
| Intercept | 255.76 | 1 | **<.001** |
| Condition | 1.46 | 1 | .23 |
| Accuracy | 2.48 | 1 | .12 |
| Age | 0.44 | 1 | .51 |
| Condition x Accuracy | 0.85 | 1 | .36 |
| Condition x Age | 0.58 | 1 | .44 |

**Table H.2**

*Relation between Response Bias and Perspective Taking Accuracy: Mixed Model Omnibus Results*

|  | $\chi$^2^ | *df* | *p*-value |
| --- | --- | --- | --- |
| Intercept | 6.26 | 1 | **.01** |
| Condition | 9.52 | 1 | **.002** |
| Accuracy | 4.24 | 1 | **.04** |
| Age | 5.16 | 1 | **.02** |
| Condition x Accuracy | 0.80 | 1 | .37 |
| Condition x Age | 4.12 | 1 | **.04** |

**Table S6**

*Summary of Model Fit Indices across all Models*

|  | **AIC** | **logLik** | $\boldsymbol{\chi}$**^2^** | ***df*** | ***p*-value** |
| --- | --- | --- | --- | --- | --- |
| **Hypothesis 1 Memory Sensitivity** |  |  |  |  |  |
| Model A1  (linear trend of age) | 403.85 | -195.93 | - | - | - |
| Model A2  (linear + quadratic trend of age) | 407.71 | -195.85 | 0.14 | 2 | .93 |
| Model A3  (linear + quadratic + cubic trend of age) | 410.69 | -195.34 | 1.02 | 2 | .60 |
| **Response Bias** |  |  |  |  |  |
| Model B1  (linear trend of age) | 116.66 | -52.33 | - | - | - |
| Model B2  (linear + quadratic trend of age) | 120.54 | -52.27 | 0.12 | 2 | .94 |
| Model B3  (linear + quadratic + cubic trend of age) | 118.97 | -49.48 | 5.57 | 2 | .06 |
| **RTs** |  |  |  |  |  |
| Model C1  (linear trend of age) | 16926 | -8452.9 | - | - | - |
| Model C2  (linear + quadratic trend of age) | 16930 | -8451.3 | 3.26 | 4 | .52 |
| Model C3  (linear + quadratic + cubic trend of age) | 16935 | -8449.5 | 3.61 | 4 | .46 |
| **Hypothesis 2 Perspective Taking Accuracy** |  |  |  |  |  |
| Model D1  (linear trend of age) | 1197.7 | -591.82 | - | - | - |
| Model D2  (linear + quadratic trend of age) | 1198.2 | -590.07 | 3.50 | 2 | .17 |
| Model D3  (linear + quadratic + cubic trend of age) | 1200.6 | -589.29 | 1.56 | 2 | .46 |
| **Perspective Taking RTs** |  |  |  |  |  |
| Model E1  (linear trend of age) | 21163 | -10575 | - | - | - |
| Model E2  (linear + quadratic trend of age) | 21165 | -10574 | 2.09 | 2 | .35 |
| Model E3  (linear + quadratic + cubic trend of age) | 21168 | -10574 | 0.58 | 2 | .75 |
| **Hypothesis 3 Memory Sensitivity Difference Scores** |  |  |  |  |  |
| Model F1  (linear trend of age) | 191.77 | -90.89 | - | - | - |
| Model F2  (linear + quadratic trend of age) | 195.58 | -90.79 | 0.08 | 2 | .91 |
| Model F3  (linear + quadratic + cubic trend of age) | 197.26 | -89.63 | 0.96 | 2 | .35 |
| **Response Bias Difference Scores** |  |  |  |  |  |
| Model G1  (linear trend of age) | 81.93 | -35.96 | - | - | - |
| Model G2  (linear + quadratic trend of age) | 82.48 | -34.23 | 0.43 | 2 | .19 |
| Model G3  (linear + quadratic + cubic trend of age) | 84.31 | -33.16 | 0.26 | 2 | .36 |
| **Exploratory** |  |  |  |  |  |
| **Learning Phase Rating**  Model X.A1  (linear trend of age) | 17315 | -8651.6 | - | - | - |
| Model X.A2  (linear + quadratic trend of age) | 17316 | -8650.2 | 2.68 | 2 | .26 |
| Model X.A3  (linear + quadratic + cubic trend of age) | 17320 | -8649.8 | 0.81 | 2 | .67 |
| **Learning Phase Reaction Times** |  |  |  |  |  |
| Model X.B1  (linear trend of age) | 5998 | -2993 | - | - | - |
| Model X.B2  (linear + quadratic trend of age) | 5994 | -2989 | 7.99 | 2 | **.02** |
| Model X.B3  (linear + quadratic + cubic trend of age) | 5997 | -2988 | 1.33 | 2 | .51 |

| **Recall Phase Rating** |  |  |  |  |  |
| --- | --- | --- | --- | --- | --- |
| Model X.C1  (linear trend of age) | 12613 | -6300.7 | - | - | - |
| Model X.C2  (linear + quadratic trend of age) | 12616 | -6300.0 | 1.31 | 2 | .52 |
| Model X.C3  (linear + quadratic + cubic trend of age) | 12618 | -6298.8 | 2.49 | 2 | .29 |
| **Recall Phase Reaction Times** |  |  |  |  |  |
| Model X.C1  (linear trend of age) | 4949 | -2469 | - | - | - |
| Model X.C2  (linear + quadratic trend of age) | 4953 | -2469 | 0.19 | 2 | .91 |
| Model X.C3  (linear + quadratic + cubic trend of age) | 4953 | -2467 | 3.59 | 2 | .17 |

*Note.* Each analysis started by assessing whether relative to the linear trend of age alone, the quadratic and cubic trends of age provided a better fit to the data. Polynomials were orthogonalized to avoid multicollinearity, and, afterwards, the model with the lowest AIC (Akaike Information Criterion) value was selected. Goodness-of-fit between models was further confirmed by comparing the likelihood of the models. If this difference was not statistically significant then the simpler model was retained. Within each outcome variable, the second row shows comparisons between model 2 and model 1, while the third row compares model 3 and model 2.

**Autistic Traits**

Autistic traits have been suggested to moderate the self-reference effect (Lombardo et al., 2007; Toichi et al., 2002). To control for autistic traits, we used the Autism Quotient (AQ) questionnaire, which was completed before the learning phase of the self-referential memory task and consists of 50 items assessing behaviors across five domains: social skills, attention switching, attention to detail, communication, and imagination. Items such as “I prefer to do things with others rather than on my own” are rated on a four-point scale (“definitely agree”, “slightly agree”, slightly disagree”, “definitely disagree”; Baron-Cohen et al., 2001). Each item scores 1 point if the respondent records autistic-like behavior (e.g., to prefer doing things on their own rather than with others) either mildly or strongly. A higher score indicates a higher level of autistic traits. Although a useful screening method to quantify where an individual lies along the dimension of autistic traits, this self-report instrument was not diagnostic and has been used previously in typical adolescents and adults (Baron-Cohen et al., 2006). There were no significant differences between adolescents and adults in the current study on the AQ (*t*(95) = -1.19, *p* = .24; see Table S7 below) and all results held after controlling for autistic traits (see Tables XA to XG below).

**Table S7**

*Autism Quotient Questionnaire: Descriptive Statistics*

|  | **Adolescents**  (*n* = 50; aged 11.2-17.5) | | |  | **Adults**  (*n* = 47; aged 22.2-35.6) | | |  |  |  |
| --- | --- | --- | --- | --- | --- | --- | --- | --- | --- | --- |
|  | *M* | *SD* | Range |  | *M* | *SD* | Range |  | *t*-value | *p*-value |
| **AQ** | 17.18 | 5.63 | 4-30 |  | 18.68 | 6.79 | 6-35 |  | -1.19 | .24 |

*Note.* Descriptive statistics of participants included in the analyses (all male) by Autism Quotient (AQ) score. M = Mean, SD = Standard deviation.

***Model XA: Memory Sensitivity***

We examined how age and condition (self vs. town) influenced memory sensitivity with a linear mixed-effects model whilst controlling for autistic traits (as measured by the Autism Quotient (AQ) questionnaire). All predictors of interest were included in the model as interacting fixed effects. The model also included random intercepts for each participant. In this model and all subsequent models, age was treated as a continuous variable for which we computed orthogonal polynomials to avoid multicollinearity.

**Table XA.1**

*Memory Sensitivity: Mixed Model Omnibus Results*

|  | $\chi$^2^ | *df* | *p*-value |
| --- | --- | --- | --- |
| Intercept | 70.34 | 1 | **<.001** |
| Age | 0.66 | 1 | .42 |
| Condition | 0.68 | 1 | .41 |
| AQ | 2.93 | 1 | .09 |
| Age x Condition | 0.27 | 1 | .60 |

***Model XB: Response Bias***

We examined how age and condition (self vs. town) influenced response bias with a linear mixed-effects model whilst controlling for autistic traits. All predictors of interest were included in the model as interacting fixed effects. The model also included random intercepts for each participant.

**Table XB**

*Response Bias: Mixed Model Omnibus Results*

|  | $\chi$^2^ | *df* | *p*-value |
| --- | --- | --- | --- |
| Intercept | 0.35 | 1 | .16 |
| Age | 8.25 | 1 | **.004** |
| Condition | 14.77 | 1 | **<.001** |
| AQ | 0.02 | 1 | .89 |
| Age x Condition | 4.73 | 1 | **.03** |

***Model XC: Memory Reaction Times***

We examined how age, condition (self vs. town), and category (target vs. distractor adjectives) influenced reaction times (RTs) for recognized adjectives with a linear mixed-effects model whilst controlling for autistic traits. RTs were log-transformed to better approximate normal distribution. All predictors of interest were included in the model as interacting fixed effects. The model also included random intercepts for each participant.

**Table XC**

*Memory Reaction Times: Mixed Model Omnibus Results*

|  | $\chi$^2^ | *df* | *p*-value |
| --- | --- | --- | --- |
| Intercept | 7638.24 | 1 | **<.001** |
| Age | 3.01 | 2 | .08 |
| Condition | 3.10 | 1 | .08 |
| Category | 12.32 | 1 | **<.001** |
| AQ | 3.42 |  | .06 |
| Age x Condition | 0.003 | 2 | .95 |
| Age x Category | 0.69 | 2 | .41 |
| Condition x Category | 1.90 | 1 | .17 |
| Age x Condition x Category | 14.10 | 2 | **<.001** |

***Model XD: Perspective Taking Accuracy***

We examined how age and trial type (experimental vs. control) influenced perspective taking accuracy with a generalized linear mixed-effects model whilst controlling for autistic traits. All predictors of interest were included in the model as interacting fixed effects. The model also included random intercepts for each participant and allowed for different slopes across trial type.

**Table XD**

*Perspective Taking Accuracy: Mixed Model Omnibus Results*

|  | $\chi$^2^ | *df* | *p*-value |
| --- | --- | --- | --- |
| Intercept | 4.34 | 1 | **.037** |
| Age | 0.80 | 1 | .3 |
| Trial type | 123.95 | 1 | **<.001** |
| AQ | 1.36 | 1 | .24 |
| Age x Trial type | 8.57 | 1 | **.003** |

***Model XE: Perspective Taking Reaction Times***

We examined how age and trial type (experimental vs. control) influenced perspective taking RTs with a linear mixed-effects model whilst controlling for autistic traits. All predictors of interest were included in the model as interacting fixed effects. The model also included random intercepts for each participant.

**Table XE**

*Perspective Taking Reaction Times: Mixed Model Omnibus Results*

|  | $\chi$^2^ | *df* | *p*-value |
| --- | --- | --- | --- |
| Intercept | 1250.66 | 1 | **<.001** |
| Age | 0.47 | 1 | .49 |
| Trial type | 21.78 | 1 | **<.001** |
| AQ | 0.33 |  | .57 |
| Age x Trial type | 0.04 | 1 | .84 |

***Model XF: Relation between Memory Sensitivity and Perspective Taking Accuracy***

With a linear regression, we examined how age and perspective taking accuracies related to memory sensitivity difference scores whilst controlling for autistic traits.

**Table XF**

*Relation between Memory Sensitivity Difference Scores and Perspective Taking Accuracy: Regression Results*

|  | *F* | *df* | *p*-value |
| --- | --- | --- | --- |
| Intercept | 2.11 | 1,87 | .15 |
| Age | 1.14 | 1,87 | .29 |
| Experimental accuracy | 1.75 | 1,87 | .19 |
| AQ | 6.27 | 1,87 | **.01** |
| Age x Experimental accuracy | 2.30 | 1,87 | .13 |

***Model XG: Relation between Response Bias and Perspective Taking Accuracy***

With a linear regression, we examined how age and perspective taking accuracies related to response bias difference scores whilst controlling for autistic traits.

**Table XG**

*Relation between Response Bias Difference Scores and Perspective Taking Accuracy: Regression Results*

|  | *F* | *df* | *p*-value |
| --- | --- | --- | --- |
| Intercept | 4.87 | 1,87 | **.03** |
| Age | 1.40 | 1,87 | .24 |
| Experimental accuracy | 0.75 | 1,87 | .38 |
| AQ | 0.53 | 1, 87 | .47 |
| Age x Experimental accuracy | 0.06 | 1,87 | .81 |

**Exploratory Analyses**

***Learning- and Recall Phase Ratings***

To examine how participants’ learning- and recall phase ratings changed as a function of age and condition, we ran two exploratory linear mixed-effects models (see Figure X1, Model X1.1 and X2.1 as well as Table X1.1 and 2.1 below). For learning phase ratings, we observed a main effect of Age, $\chi$^2^ (1) = 8.75, *p* = .003, η_p_^2^ = .09, a main effect of Condition, $\chi$^2^ (1) = 175.75, *p*< .001, η_p_^2^ = .65, but no significant Age x Condition interaction, $\chi$^2^ (1) = 2.28, *p* = .13 (see Figure X1A below). For recall phase ratings, we observed no main effect of Age, $\chi$^2^ (1) = 3.27, *p* = .07, a significant main effect of Condition, $\chi$^2^ (1) = 7.31, *p* = .007, η_p_^2^ = .07, and a significant Age x Condition interaction effect, $\chi$^2^ (1) = 6.34, *p* = .01, η_p_^2^ = .06 (see Figure X1B below). Post-hoc contrasts within the model suggested that the interaction between age and condition was driven by lower confidence ratings for town-related target adjectives than for self-related target adjectives, contrast _town(target) – self(target)_ = -0.11, SE = .04, *p*_Bonf_ = .007. However, this tendency decreased linearly with age, slope _town(target)_ = 0.02, SE = .01, *p*_Bonf_ = .02. In other words, younger adolescents were less confident in recalling town-related target adjectives than were adults.

***Model X1.1: Learning Phase Ratings***

We examined how age and condition (self vs. town) influenced learning phase ratings with a linear mixed-effects model. All predictors of interest were included in the model as interacting fixed effects. The model also included random intercepts for each participant.

**Table X1.1**

*Learning Phase Ratings: Mixed Model Results*

|  | $\chi$^2^ | *df* | *p*-value |
| --- | --- | --- | --- |
| Intercept | 6469.42 | 1 | **<.001** |
| Age | 8.75 | 1 | **.003** |
| Condition | 175.75 | 1 | **<.001** |
| Age x Condition | 2.28 | 1 | .13 |

***Model X2.1: Recall Phase Ratings***

We examined how age and condition (self vs. town) influenced recall phase ratings with a linear mixed-effects model. All predictors of interest were included in the model as interacting fixed effects. The model also included random intercepts for each participant.

**Table X2.1**

*Recall Phase Ratings: Mixed Model Results*

|  | $\chi$^2^ | *df* | *p*-value |
| --- | --- | --- | --- |
| Intercept | 10393.18 | 1 | **<.001** |
| Age | 3.27 | 1 | .07 |
| Condition | 7.31 | 1 | **.007** |
| Age x Condition | 6.34 | 1 | **.01** |

**Figure X1**

*Learning- and Recall Phase Ratings in the Self-Referential Memory Task*


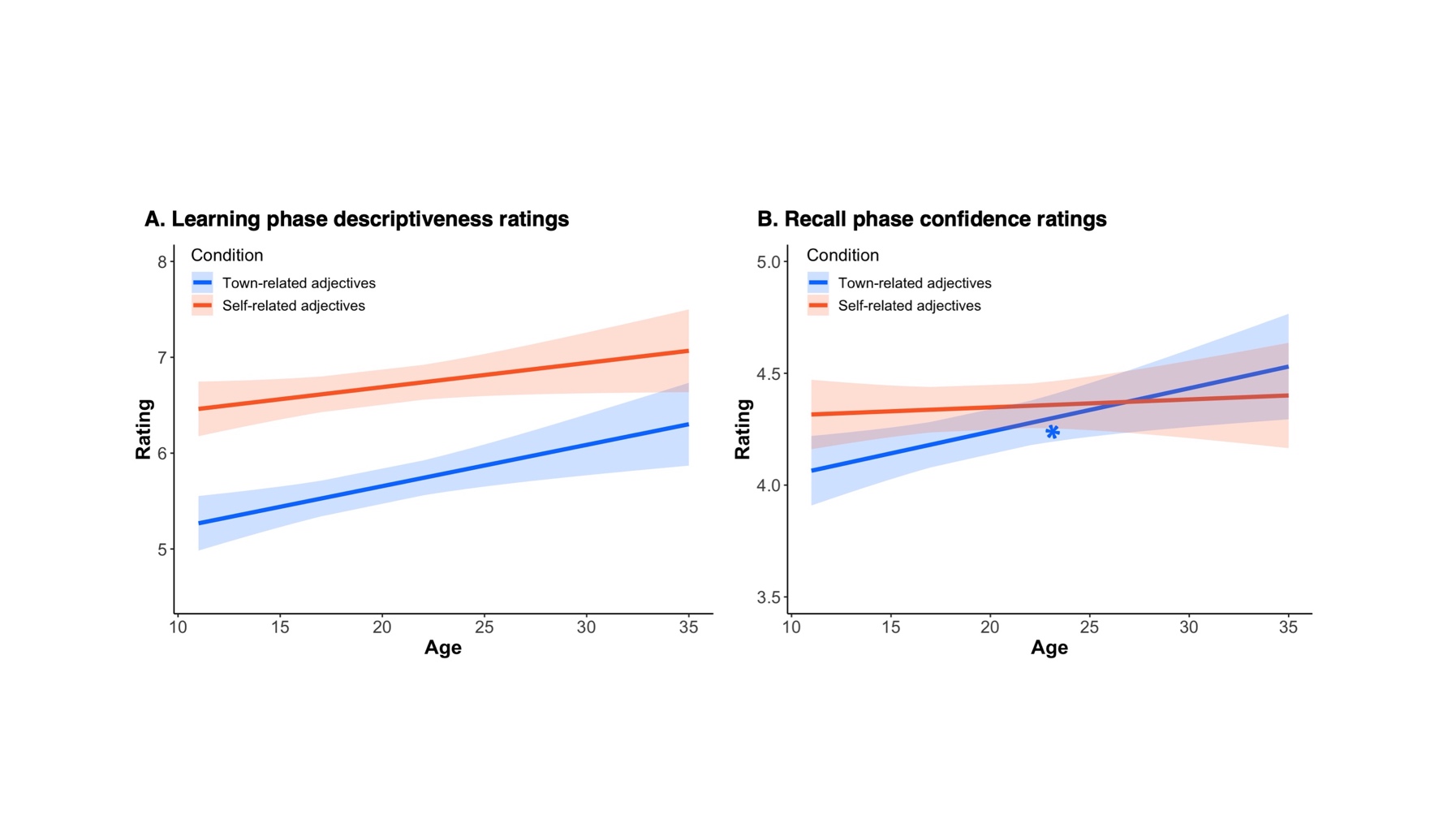


*Note*. (**A**) Effect of age on learning phase descriptiveness ratings. Mean learning phase descriptiveness ratings (y-axis) are plotted as a function of age (x-axis) for each condition. (**B**) Effect of age on recall phase confidence ratings. Mean recall phase confidence ratings (y-axis) are plotted as a function of age (x-axis) for each condition. Across age, participants were similarly confident in recalling self-related target adjectives. However, younger adolescents were less confident in recalling town-related target adjectives than adults (slope _town(target)_ = 0.02, SE = .01, *p*_Bonf_ = .02). All colored lines and shaded .95 CIs show the linear trends as estimated by the trial-level linear mixed-effects models. * *p*_Bonf_< .05.

***Learning- and Recall Phase Reaction Times***

To examine how participants’ learning- and recall phase reaction times changed as a function of age and condition, we ran two exploratory linear mixed-effects models (see Figure X2, Model X1.2 and 2.2 as well as Table X1.2 and 2.2). For learning phase reaction times, we observed a main effect of Age-squared, $\chi$^2^ (2) = 10.87, *p* = .004, η_p_^2^ = .10, a main effect of Condition, $\chi$^2^ (1) = 20.18, *p*< .001, η_p_^2^ = .18, and a significant Age-squared x Condition interaction, $\chi$^2^ (2) = 18.24, *p*< .001, η_p_^2^ = .16 (see Figure X2A below). Post-hoc contrasts within the model suggested that the interaction between age-squared and condition was driven by slower reaction times for town-related target adjectives than for self-related target adjectives, contrast _town(target) – self(target)_ = 0.02, SE = .01, *p*_Bonf_ = .01. This tendency was best explained by a quadratic trend of age, slope _town(target)_ = 0.02, SE = .01, *p*_Bonf_ = .01. For recall phase reaction times, we observed no main effect of Age, $\chi$^2^ (1) = 2.97, *p* = .08, no significant main effect of Condition, $\chi$^2^ (1) = 0.29, *p* = .59, but a significant Age x Condition interaction effect, $\chi$^2^ (1) = 5.07, *p* = .02, η_p_^2^ = .05 (see Figure X2B below). Post-hoc contrasts within the model suggested that the interaction between age and condition was drive by slower reaction times for town-related target adjectives than for self-related target adjectives, _town(target) – self(target)_ = 0.01, SE = .003, *p*_Bonf_< .001. However, this tendency decreased linearly with age, slope _town(target)_ = -0.01, SE = .004, *p*_Bonf_ = .047.

***Model X1.2: Learning Phase Reaction Times***

We examined how age and condition (self vs. town) influenced learning phase reaction times with a linear mixed-effects model. All predictors of interest were included in the model as interacting fixed effects. The model also included random intercepts for each participant. Please note that this model included the second-degree polynomial of age (i.e., including linear and quadratic functions of age).

**Table X1.2**

*Learning Phase Reaction Times: Mixed Model Results*

|  | $\chi$^2^ | *df* | *p*-value |
| --- | --- | --- | --- |
| Intercept | 44473.19 | 1 | **<.001** |
| Age-squared | 10.87 | 2 | **.004** |
| Condition | 20.18 | 1 | **<.001** |
| Age-squared x Condition | 18.23 | 2 | **<.001** |

***Model X2.2: Recall Phase Reaction Times***

We examined how age and condition (self vs. town) influenced recall phase reaction times with a linear mixed-effects model. All predictors of interest were included in the model as interacting fixed effects. The model also included random intercepts for each participant.

**Table X2.2**

*Recall Phase Reaction Times: Mixed Model Results*

|  | $\chi$^2^ | *df* | *p*-value |
| --- | --- | --- | --- |
| Intercept | 84402.24 | 1 | **<.001** |
| Age | 2.97 | 1 | .08 |
| Condition | 0.29 | 1 | .59 |
| Age x Condition | 5.07 | 1 | **.02** |

**Figure X2**

*Learning- and Recall Phase Reaction Times in the Self-Referential Memory Task*


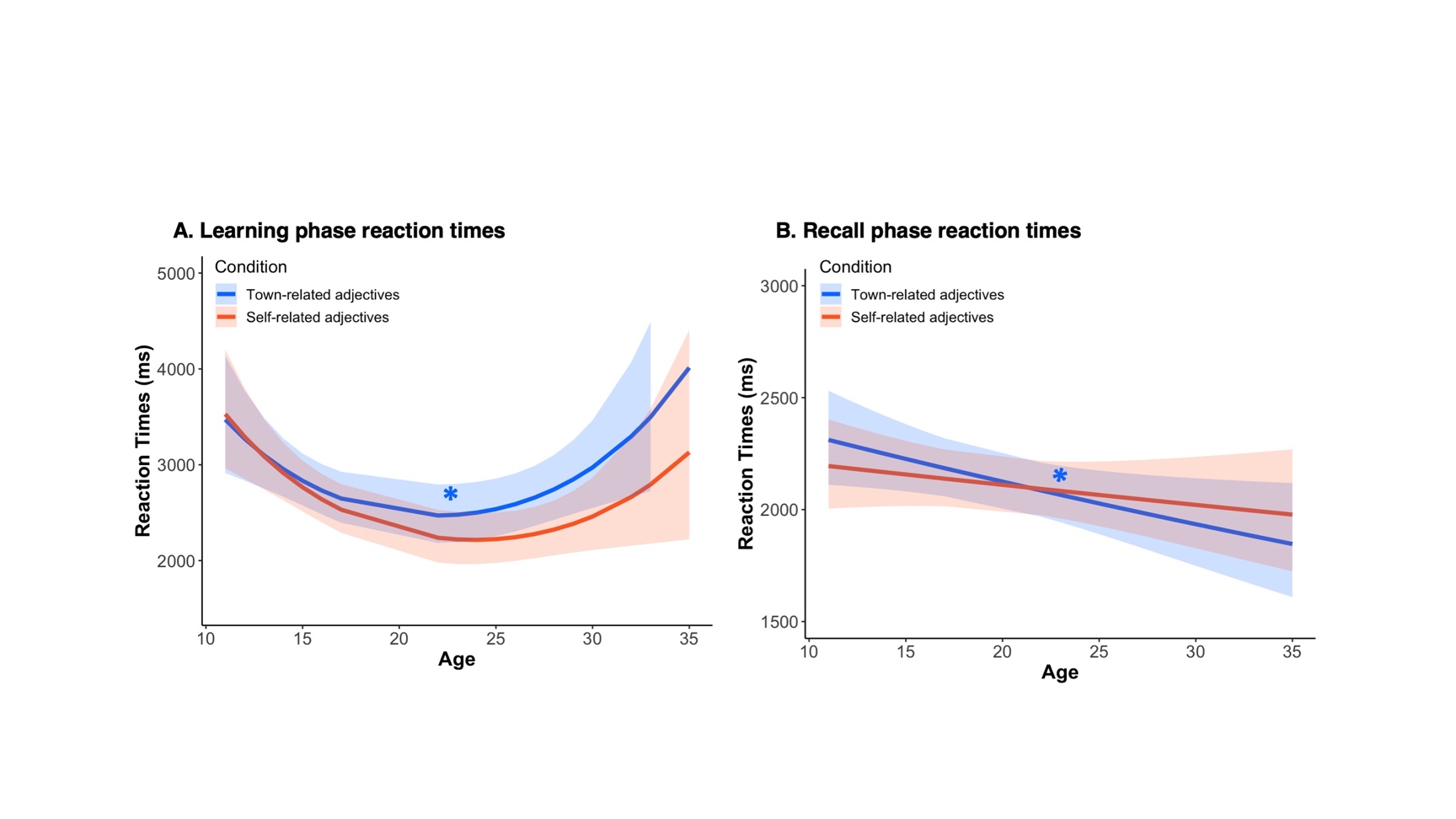


*Note*. (**A**) Effect of age on learning phase reaction times. Median learning phase reaction times (y-axis) are plotted as a function of age squared (x-axis) for each condition. (**B**) Effect of age on recall phase reaction times. Median recall phase reaction times (y-axis) are plotted as a function of age (x-axis) for each condition. Across age, participants were slower when rating town-related adjectives during the learning phase (slope _town(target)_ = 0.02, SE = .01, *p*_Bonf_ = .01), however, this tendency decreased linearly with age (slope _town(target)_ = -0.01, SE = .004, *p*_Bonf_ = .047). All colored lines and shaded .95 CIs show the quadratic (learning phase) or linear trends (recall phase) as estimated by the trial-level linear mixed-effects models.
* *p*_Bonf_< .05.
